# Transverse spin dynamics in structured electromagnetic guided waves

**DOI:** 10.1073/pnas.2018816118

**Published:** 2021-02-01

**Authors:** Peng Shi, Luping Du, Congcong Li, Anatoly V. Zayats, Xiaocong Yuan

**Affiliations:** ^a^Nanophotonics Research Center, Shenzhen Key Laboratory of Micro-Scale Optical Information Technology & Institute of Microscale Optoelectronics, Shenzhen University, Shenzhen 518060, China;; ^b^Department of Physics, King’s College London, London WC2R 2LS, United Kingdom;; ^c^London Centre for Nanotechnology, King’s College London, London WC2R 2LS, United Kingdom

**Keywords:** angular momentum of light, spin–orbit coupling, transverse spin, spin–momentum locking

## Abstract

Spin–momentum locking, a manifestation of topological properties that governs the behavior of surface states, was studied intensively in condensed-matter physics and optics, resulting in the discovery of topological insulators and related effects and their photonic counterparts. In addition to spin, optical waves may have complex structure of vector fields associated with orbital angular momentum or nonuniform intensity variations. Here, we derive a set of spin–momentum equations which describes the relationship between the spin and orbital properties of arbitrary complex electromagnetic guided modes. The predicted photonic spin dynamics is experimentally verified with four kinds of nondiffracting surface structured waves. In contrast to the one-dimensional uniform spin of a guided plane wave, a two-dimensional chiral spin swirl is observed for structured guided modes. The proposed framework opens up opportunities for designing the spin structure and topological properties of electromagnetic waves with practical importance in spin optics, topological photonics, metrology and quantum technologies and may be used to extend the spin-dynamics concepts to fluid, acoustic, and gravitational waves.

## Significance

We formulate and experimentally validate a set of spin–momentum equations which are analogous to the Maxwell’s equations and govern spin–orbit coupling in electromagnetic guided waves. The Maxwell-like spin–momentum equations reveal the spin–momentum locking, the chiral spin texture of the field, Berry phase, and the spin–orbit interaction in the optical near field. The observed spin–momentum behavior can be extended to other classical waves, such as acoustic, fluid, gas, and gravitational waves.

Spin–momentum locking, characterized by unidirectional surface spin states, has been extensively studied in topological insulators (1), superconductors (2), magnons (3), and cold-atom (4) and Bose–Einstein condensates (5). The photonic analogy of unidirectional surface spin states was demonstrated with the pseudospin by engineering an “extrinsic” spin–orbit interaction and breaking the time-reversal symmetry in artificial photonic structures (6–8). On the other hand, due to an “intrinsic” spin–orbit coupling governed by the Maxwell’s field theory, the spin–momentum locking of light was reported and linked to the modes with the evanescent field components, such as surface waves or waveguided modes (9–11). For example, surface plasmon polaritons (SPPs) as surface modes propagating at an insulator–metal interface (12) exhibit features of spin–momentum locking that are analogous to the behavior of surface state of a topological insulator (6–8). Although photons are bosons with integer spin and surface and waveguided electromagnetic modes suffer from backscattering (13), in contrast to the helical fermion behavior of surface Dirac modes, they possess the topological ℤ_4_ invariant and hence can transport spin unidirectionally (9). This intrinsic optical spin–momentum locking is a basis for many intriguing phenomena such as spin-controlled unidirectional excitation of surface and waveguided modes and offers potential applications in photonic integrated circuits, polarization manipulation, metrology, and quantum technologies for generating polarization entangled states (14–20).

Optical transverse spin plays a key role in the intrinsic spin–momentum locking effect in evanescent waves (11). In contrast to a conventional, longitudinal spin of light with the spin vector parallel to the propagating direction, the orientation of transverse spin is perpendicular to the propagating direction, enabling many important phenomena and applications (21–28). An empirical procedure to identify the optical spin direction includes calculating the spin angular momentum (SAM) **S** and comparing the spin orientation to the wave vector **k**. This empirical perspective provides an intuitive way to identify the optical transverse spin in various optical configurations involving plane waves but cannot be generalized to more complex scenarios, for example when structured waves with an arbitrary trajectory and orbital angular momentum need to be considered. Although one can define a “local” wave vector, which is related to the orbital energy flow density **P**_o_, it cannot describe quantitatively an optical transverse spin associated with a structured vector wave for which the spin part of the Poynting vector (**P**_s_) is also important (29).

Here, we overcome this limitation and extend the understanding of the spin–momentum locking and spin dynamics from plane evanescent waves to a two-dimensional (2D) chiral spin swirl associated with the structured guided modes, therefore generalizing the optical spin–momentum locking to arbitrary guided vector fields. From the perspective of energy flow density (**P** = **P**_s_ + **P**_o_), we derive four equations describing dynamic transformations of spin and momentum of the electromagnetic wave that are analogous to the Maxwell’s equations for electromagnetic fields. The proposed framework is verified experimentally on the example of four structured surface waves and opens up opportunities for understanding and designing the spin dynamics and topological properties of electromagnetic waves from the radiofrequency to ultraviolet spectral ranges and for applications in spin optics, topological photonics, polarization measurements, metrology, and quantum technologies. Since the energy flow density can be represented through a current density term in the Hertz potential (*SI Appendix*, section VI), the proposed description allows also extending the concepts of the dynamics of transverse spin from electromagnetic waves to fluid, acoustic, and gravitational waves (30–32).

## Results and Discussion

For an arbitrary electromagnetic wave propagating in a homogeneous medium, the curl of the energy flow density can be presented as (*SI Appendix*, section I)

∇×Ρ=v2∇×p=ω2S−14Re{−(∇⊗E∗)⋅H−(∇⊗E)Τ⋅H∗+(∇⊗H∗)⋅E+(∇⊗H)Τ⋅E∗}, [1]

where **p** is the kinetic momentum density of the field, which is linearly related to the Poynting vector in a homogeneous medium **p** = **P**/*v*^2^, *v* is the speed of light in the medium, *ω* represents the angular frequency of the electromagnetic field, and **E** and **H** indicate the electric and magnetic field, respectively. Here, the symbol ⊗ indicates a dyad vector and ∗ denotes the complex conjugate. The second part on the right-hand side of Eq. **1** has the same structure as the quantum 2-form (33) that generates the Berry phase associated with a circuit, which indicates a spin–orbit interaction in the optical system (*SI Appendix*, section II). In particular, for electromagnetic waves with an evanescent field, such as surface or guided waves, an intrinsic spin–momentum relationship can be derived from the Maxwell’s theory:

$$S=\frac{1}{2\omega^{2}}\nabla \times P=\frac{1}{2k^{2}}\nabla \times p,$$ [2]

where *k = ω/v* is the wave number of the electromagnetic wave in the medium. Since curl of a vector field can be regarded as its current vortices, Eq. **2** reveals that the optical spin of an evanescent field is associated with the local vorticity of the electromagnetic energy flow density and is source-less (∇·**S** = 0). The SAM in this case is related to the transverse gradient of the energy flow density. At the same time, the longitudinal optical spin does not fulfill the above spin–momentum relationship. For example, a monochromatic circularly polarized plane wave bears the SAM aligned parallel to the wave vector, while the curl of the Poynting vector vanishes because of the uniformity of the energy flow density over the space. Therefore, the spin–momentum law in Eq. **2** only describes the dynamics of optical transverse spin present in the evanescent waves. It also reveals that, in addition to the optical spin oriented along the surface (in-plane transverse SAM), which has been recently studied intensively, there exists another category of the transverse spin of an evanescent field oriented out of the surface plane. This SAM can be induced by the in-plane energy flow density of the structured guided or surface wave, while the in-plane transverse spin is due to the gradient of energy flow density normal to the interface. The appearance of a transverse spin indicates to the rotation of polarization and hence the phase difference between all the field components of the wave.

**Fig. 1.**
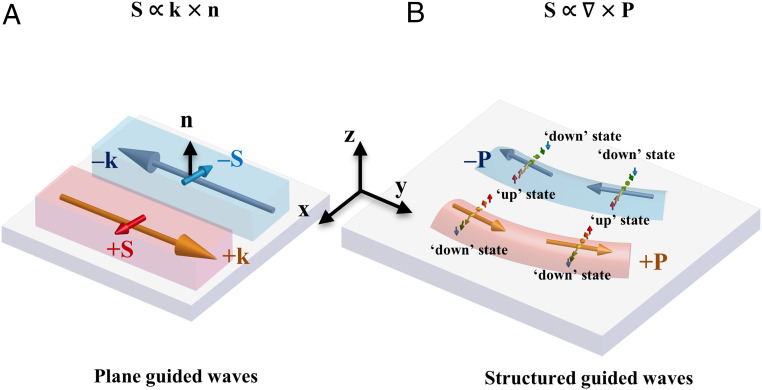
Generalization of spin–momentum locking for structured guided modes. (*A*) In unstructured, plane guided wave, optical spin–momentum locking results in the transverse spin (S) uniformly distributed and parallel to the interface. The spin vector direction is perpendicular to the wave vector **k** and flips if the propagation direction flipped from +**k** to −**k**. (*B*) In an arbitrary structured guided wave, the optical spin is related to the vorticity of the energy flow density **P**. The transverse spin vector varies from the “up” state to the “down” state around the energy flow density, remaining perpendicular to the local wave vector. This forms a chiral swirl of the 2D transverse spin which is locked to the energy propagating direction and fulfills a right-handed rule. The direction of the local transverse spin vector flips if the energy flow density flipped from forward (+**P**) to backward (−**P**).

The spin–momentum locking in an evanescent plane wave (Fig. 1*A*) as demonstrated in previous work (9) is a special, one-dimensional (1D) case of spin–momentum locking with the SAM vector aligned along the interface. Assuming the guided mode propagating along the y direction and evanescently decaying along the z direction, one can deduce the Poynting vector **P** = $\hat{y}$*ωε*/(2*β*)exp(−2*k*_*z*_*z*) and the SAM **S** = $\hat{x}$*εk*_*z*_/(2*ωβ*)exp(−2*k*_*z*_*z*), where *ε* denotes the permittivity of the medium and *β* and *ik*_*z*_ stand for the in-plane and out-of-plane wave vector components, respectively. The energy flow density and SAM of the evanescent plane wave are connected through the generalized spin–momentum relation: **S** = ∇ × **P**/(2*ω*^2^) = ‒$\hat{x}$(∂*P*_y_/∂*z*)/(2*ω*^2^). For structured evanescent modes with spatially varying intensity distribution, the inhomogeneity of energy flow density can induce several SAM components in different directions. The variation of the energy flow density in the z direction induces an in-plane component of the SAM, while its in-plane variations induce a z component. Both are perpendicular to the local energy propagation direction. The relationship between the two components leads to a chiral spin texture with spin vectors swirling around the energy flow (Fig. 1*B*). More importantly, its tendency of directional variation (i.e., the chirality) is locked to the momentum direction. This is a manifestation of the generalized optical spin–momentum locking associated with an electromagnetic evanescent wave.

It should be noted that the transverse spin discussed here is different from the “spins” in conventional topological photonics, typically called a “pseudospin.” For a pseudospin, the spin–momentum locking is achieved by engineering the spin–orbit interaction in artificial photonic structures in order to break the time-reversal symmetry (8). In the case of the optical transverse spin of an evanescent wave, the generalized spin–momentum locking is an “intrinsic” feature of the spin–orbit interaction governed solely by the Maxwell’s theory. The nonzero spin Chern number for the structured waves (*SI Appendix*, section IV) implies the existence of nontrivial helical states of electromagnetic waves which are strictly locked to the energy propagation direction. However, since the topological ℤ_2_ invariant of these states vanishes owing to the time-reversal symmetry of the Maxwell’s equations, there is no protection against (back)scattering. Although the transformation of the two helical states of evanescent waves is not topologically protected against scattering, the spin–momentum locking and the induced unidirectional excitation and propagation are the intrinsic feature of the Maxwell’s theory and are topologically nontrivial, possessing the ℤ_4_ topological invariant.

**Fig. 2.**
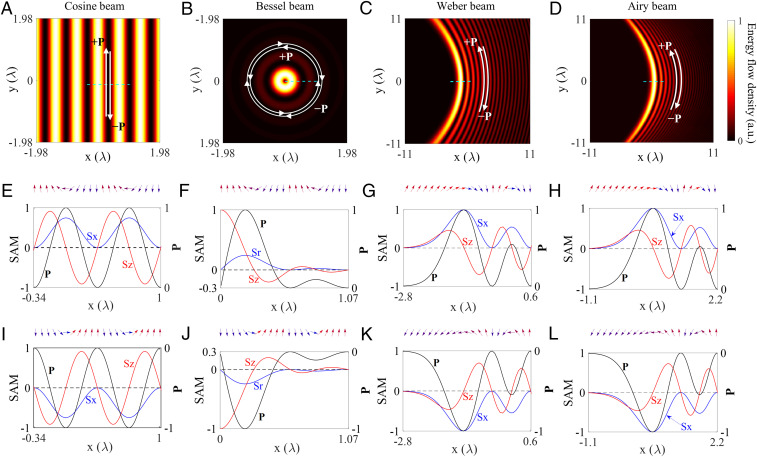
Spin–momentum locking in various surface structured waves. (*A*–*D*) The spatial distributions of the energy flow density for different structured surface waves: (*A*) surface Cosine beam, (*B*) surface Bessel beam with topological charge l = ±1, (*C*) surface Weber beam, and (*D*) surface Airy beam. These beams can either propagate in the forward (labeled +**P**) or backward (labeled –**P**) directions. (*E*–*H*) Transverse SAM components Sz and Sx and the cross-sections of the energy flow density distributions along the green dashed lines in *A*–*D* for the beams propagating in the direction indicated with the arrow labeled +**P**. (*I*–*L*) The same as *E*–*H* for the beams propagating in the opposite direction indicated with the arrow labeled –**P** in *A*–*D*. The inserts at the top of *E*–*L* show the local transverse spin vector orientations (cf. Fig. 1). The spin vectors are swirling around the energy flow density and their local orientations vary from the “up” to the “down” states, fulfilling the right-handed rule. These orientations are seen inverted for the waves with the opposite direction of the energy propagation. Note that for the beams with curved trajectory, the spin variation is considered in the plane perpendicular to the local tangential direction of the energy flow density. The distance unit is the wavelength of light in vacuum.

To demonstrate the spin–momentum locking features described by Eq. **2**, four types of the electromagnetic modes exhibiting evanescent field with inhomogeneous spatial energy distribution were investigated, including the solutions of a wave equation in Cartesian coordinates (Cosine beam) (34), in cylindrical coordinates (Bessel beam) (35), in parabolic coordinates (Weber beam) (36), and in Cartesian coordinates but with a parabolic path (Airy beam) (37) (*SI Appendix*, section V). The spatial distributions of their energy flow densities are shown in Fig. 2 *A*–*D*, while the beams’ propagation directions can either be forward (+**P**) or backward (–**P**). The corresponding cross-sections along the dashed lines are shown in Fig. 2 *E*–*H* and *I–L* for the beams with opposite propagation directions, respectively, together with the SAM distributions and the spin vector variations. For all four different types of the beams, the spin orientation varies progressively from the “up” state to the “down” state when the energy propagates along the forward direction (Fig. 2 *E*–*H*). The intrinsic spin–momentum locking present in evanescent waves ensures the topological protection in terms of spin vector swirl being completely determined by the energy flow density. Thus, to observe the reversal of the spin swirling from the “down” state to the “up” state, the propagation direction must be reversed (Fig. 2 *I*–*L*). This spin–momentum locking is preserved even for surface modes suffering from the ohmic losses (12), which influence only the intensity of the wave but not the orientation of photonic spin vector. Note that the spin vector has orientation along the interface at the maxima of the energy flow density and is normal to it at the nodes. Therefore, a period of spin variation can be defined between the two adjacent nodes of energy flow density which exhibits a similar feature to a topological soliton (38–42).

**Fig. 3.**
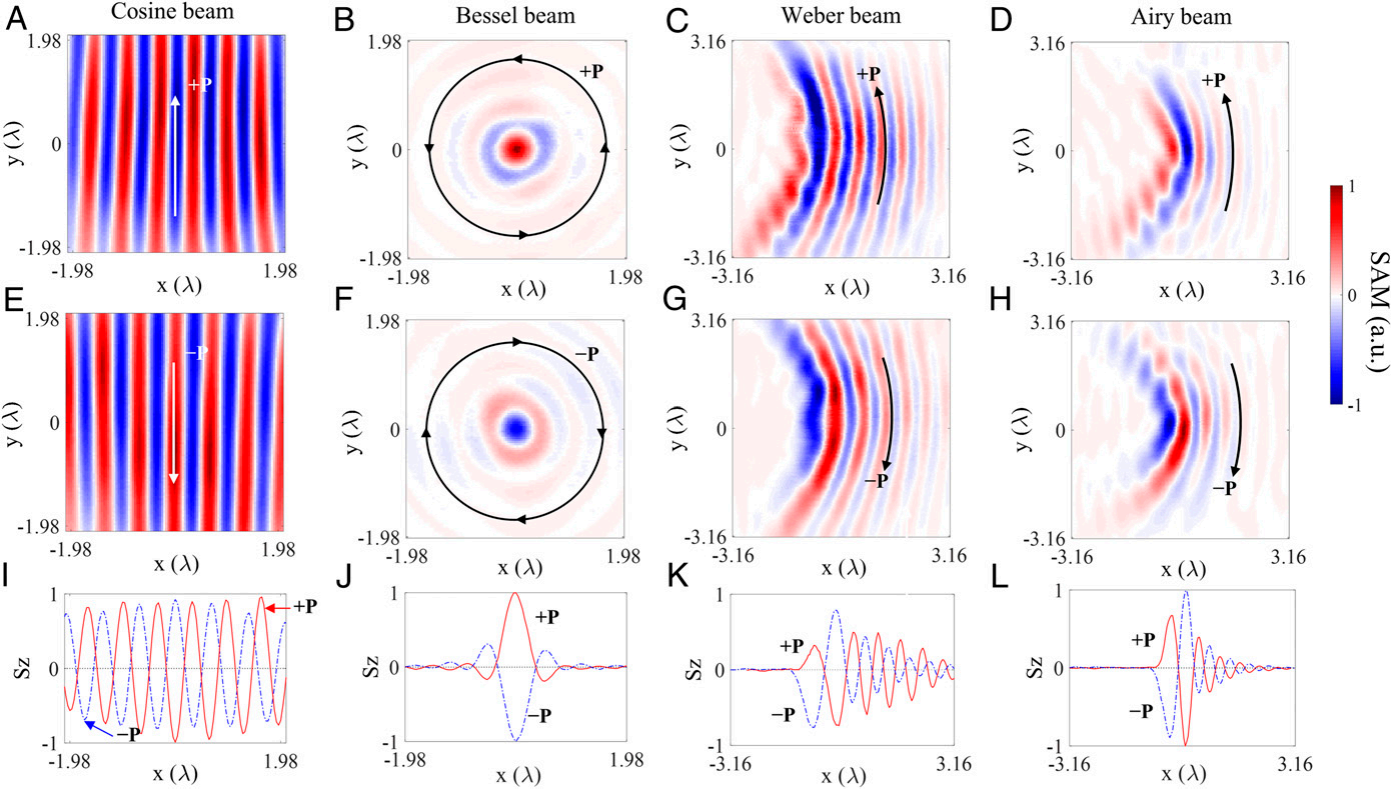
Experimental validation of the spin–momentum locking. The measured out-of-plane SAM components (S_z_) for (*A*, *E*, and *I*) surface Cosine beam, (*B*, *F*, and *J*) surface Bessel beam, (*C*, *G*, and *K*) surface Weber beam and (*D*, *H*, and *L*) surface Airy beam: the spatial distributions of *S*_*z*_ spin components for the beams with (*A*–*D*) forward (+**P**) and (*E*–*H*) opposite (−**P**) energy propagating direction, (*I*–*L*) the cross-sections of *A*–*H*. The direction of the out-of-plane transverse SAM is seen inverted for the waves propagating in opposite directions. The distance unit is the wavelength of light in vacuum.

In order to experimentally observe the spin–momentum locking features associated with the structured surface waves and out-of-plane transverse SAM, the experiments were performed on the example of SPPs (*SI Appendix*, section VII). SPPs were excited under the condition of a total internal reflection using a microscope objective with high numerical aperture (NA) = 1.49. Spatial light modulator and amplitude masks were employed to modulate the phase and wavevector of the excited SPPs to generate the desired plasmonic modes. A scanning near-field optical microscope, which employs a dielectric nanosphere to scatter the SPPs to the far field, and a combination of a quarter waveplate and a polarizer to extract the two circular polarization components (*I*_*RCP*_ and *I*_*LCP*_) of the far-field signal, were used to measure the out-of-plane SAM component *S*_*z*_ = *εβ*^2^/[4*ωk*_*z*_^2^(*I*_*RCP*_ ‒ *I*_*LCP*_)]. The corresponding in-plane spin components were also reconstructed from the measurements (*SI Appendix*, section VIII). The measured distributions of the SAM components are shown in Fig. 3 and *SI Appendix*, Figs. S16–S19 for the four types of structured SPP waves propagating in the forward and backward directions. All the predicted SAM and spin–momentum locking features are observed experimentally: 1) the variation of SAM from the positive/negative state to the negative/positive across the beam profile and 2) the reversal of spin variation when inversing the beam propagation direction.

Since the kinetic momentum density can be divided into the spin (**p**_s_) and orbital (**p**_o_) parts, **p** = **p**_s_ + **p**_o_, where **p**_s_ = ∇ × **S**/2, and obeys the spin–momentum relationship (Eq. **2**), we can formulate a set of the Maxwell-like equations linking the transverse spin and the momentum density (and the Poynting vector) of evanescent electromagnetic fields (Table 1). This formulation provides comprehensive and intuitive understanding of the boundary conditions and the dynamical properties of the spin, momentum, and energy flow in guided waves (*SI Appendix*, section III). For example, the flip of the out-of-plane spin and the in-plane Poynting vector of a SPP wave across the metal/dielectric interface immediately follows from the boundary conditions due to the opposite sign of the permittivities on the different sides of the guiding interface. The same as variations of **E** field induces **H** field in the Maxwell’s equations, equation ∇ × **p** = 2*k*^2^**S** indicates that the spatial variations of the momentum/energy flow density induce the transverse SAM. In the same manner, equation ∇ × **S** = 2**p**_s_ = 2(**p** − **p**_o_) tells us that the spin variation in turn contributes to the momentum/energy flow density, with the remainder provided from the orbital part (**p**_o_). Consolidating spin–momentum equations results in an analog of a Helmholtz equation ∇^2^**S** + 4*k*^2^**S** = 2∇ × **p**_o_, which describes spin–orbit interaction in evanescent waves, linking transverse spin and orbital part momentum density. In both the Helmholtz equation and the last Maxwell’s equation in Table 1, current **J** is an external source of magnetic field; similarly, in the corresponding spin–momentum equations, **p**_o_, which determines the orbital angular momentum, influences the spin. Since an electromagnetic wave in a source-free and homogenous medium can be described with Hertz potential (Ψ) satisfying the Helmholtz equation, and the Poynting vector can be calculated from the Hertz potential as **P** ∝ (Ψ*∇Ψ−Ψ∇Ψ*) (43), one can obtain the spin and orbital properties of the electromagnetic guided waves directly from the spin–momentum equations without any knowledge on the electric and magnetic fields (*SI Appendix*, section VI).

**Table 1.** Spin–momentum equations and the analogy to the Maxwell’s equations

Maxwell's equations	Spin–momentum equations
∇⋅E=0	∇⋅p=0
∇⋅H=0	∇⋅S=0
∇×E=iωμH	$\nabla \times p=2k^{2}S$
∇×H=J−iωεE	$\nabla \times S=2\left(p-p_{\text{o}}\right)$
Helmholtz equation	
$\nabla^{2}H+k^{2}H=-\nabla \times J$	$\nabla^{2}S+4k^{2}S=2\nabla \times p_{\text{o}}$

## Conclusion

We have demonstrated an intrinsic spin–momentum law which governs the transverse spin dynamics of guided electromagnetic waves. It was shown that the 1D uniform spin of surface plane wave evolves in a 2D chiral spin swirl for structured guided modes, providing a manifestation of the generalized photonic spin–momentum locking. Four types of structured surface waves, including the Cosine beam, Bessel beam, Weber beam, and Airy beam, have been investigated both theoretically and experimentally to demonstrate the concept of the generalized spin–momentum locking. Applying this spin-momentum locking, we obtained a set of spin–momentum equations that are analogous to the Maxwell’s equations and the boundary conditions. This optical spin framework can be used to evaluate the spin–orbit coupling in the electromagnetic guided waves and for designing specific transverse spin structures, without a priori information on the electric and magnetic fields. The generalized intrinsic spin–momentum features could also appear in other types of waves with evanescent field, such as fluid, surface elastic, acoustic, and gravitational waves. The effect could be of importance to the development of spin optics for quantum technologies and topological photonics.

## Materials and Methods

### Experimental Setup.

The experimental setup for studies of the optical spin–momentum locking is shown in *SI Appendix*, Fig. S9. The experiment was performed on the example of SPPs, which are TM-mode evanescent waves supported on a metal–dielectric interface. An He–Ne laser beam with a wavelength of 632.8 nm was used as a light source. After a telescope system to expand the beam, a combination of linear polarizer, half-wave plates, quarter-wave plates, and vortex wave plates was employed to modulate the polarization of the laser beam. A spatial light modulator was then utilized to modulate the phase of the beam. The structured beam was then tightly focused by an oil-immersion objective (Olympus, NA = 1.49, 100×) onto the sample consisting of a thin silver film (45-nm thickness) deposited on a coverslip, to form the desired SPP beams at the air/silver interface.

A polystyrene nanosphere was immobilized on the silver film surface, as a near-field probe to scatter the SPPs to the far field. The sample was fixed on a piezo scanning stage (P-545; Physik Instrumente) providing resolution down to 1 nm. A low-NA objective (Olympus, NA = 0.7, 60×) was employed to collect the scattering radiation from the nanosphere. A combination of quarter-wave plate and linear polarizer was used to extract the right-handed (RCP) and left-handed (LCP) circular polarization components of the collected signals. Finally, the intensities of RCP and LCP components are measured by a photomultiplier tube (R12829; Hamamatsu). As the piezo scanning stage raster scanned the near-field region, the distributions of RCP and LCP components can be mapped and used to reconstruct the longitudinal SAM component.

### Numerical Simulation.

The numerical simulations were performed with a custom program in MATLAB. The details can be found in *SI Appendix*, sections V, VII, and VIII.

## Supplementary Material

Supplementary File

## Data Availability

All study data are included in the article and/or *SI Appendix*.
