# Supplementary material for: Transverse spin dynamics in structured electromagnetic guided waves
Source: Proc Natl Acad Sci U S A. 2021 Feb 1;118(6):e2018816118. doi: 10.1073/pnas.2018816118 (PMC8017982; doi:10.1073/pnas.2018816118)
Supplement: Supplementary File [file pnas.2018816118.sapp.pdf]

PNAS
www.pnas.org

Supplementary Information for

**Transverse spin dynamics in structured electromagnetic guided waves**

Peng Shi[a,1], Luping Du [a,1,2], Congcong Li[a], Anatoly V. Zayats [b,2], and Xiaocong Yuan [a,2]

[a] Nanophotonics Research Center, Shenzhen Key Laboratory of Micro-Scale Optical Information Technology & Institute of Microscale Optoelectronics, Shenzhen University, Shenzhen, China

[b]Department of Physics and London Centre for Nanotechnology, King's College London, London, WC2R 2LS, United Kingdom

[1]Peng Shi and Luping Du contributed equally to this work.
[2]To whom correspondence may be addressed. Email: lpdu@szu.edu.cn, a.zayats@kcl.ac.uk or xcyuan@szu.edu.cn.

**This PDF file includes:**

Supplementary text I to VIII
Figures S1 to S19
Table S1
Equations S1-S108
SI References s1-s17

Supplementary Information Text

## I. Spin and orbit decomposition of an arbitrary electromagnetic field

All the definitions of physical quantities in the manuscript and Supplementary Materials are derived from the Maxwell's theory [s1] and the Riemann–Silberstein (R-S) vector representation for the electromagnetic fields [s2-s4]. In the following, we only consider the Cartesian coordinates (*x*, *y*, *z*) with directional unit vector $(\hat{\mathbf{x}},\hat{\mathbf{y}},\hat{\mathbf{z}})$, while the complex beams in other coordinate systems can also be derived with the same procedure.

The energy flow density, also known as the Poynting vector (**P**) of an optical wave can be expressed as [s5]:

$$\mathbf{P}=\frac{1}{2}\mathrm{Re}\left\{\mathbf{E}^{*}\times\mathbf{H}\right\}, \tag{S1}$$

where **E** and **H** represent the electric and magnetic fields, respectively. The superscript * denotes the complex conjugate. For a time-harmonic, monochromatic electromagnetic wave in the homogeneous medium, the Poynting vector can also be expressed as the momentum density of the field

$$\mathbf{p}=\mathbf{P}/v^{2}=\varepsilon\mu\mathbf{P}=\frac{1}{4\omega}\mathrm{Im}\left\{\underbrace{\varepsilon\left[\mathbf{E}^{*}\times\left(\nabla\times\mathbf{E}\right)\right]}_{Electric\leftrightarrow\mathbf{p}^{e}}+\underbrace{\mu\left[\mathbf{H}^{*}\times\left(\nabla\times\mathbf{H}\right)\right]}_{magnetic\leftrightarrow\mathbf{p}^{m}}\right\}. \tag{S2}$$

where *ε* and *μ* denote the absolute permittivity and permeability of the medium, respectively, $v=1/\sqrt{\varepsilon\mu}$ is the speed of light in a medium, and *ω* is the angular frequency of wave. The momentum density can be represented as the contributions from the electric ($\mathbf{p}^e$) and magnetic ($\mathbf{p}^m$) field components.

By considering the Gaussian's law of the electric and magnetic fields in a passive and lossless medium, the momentum density of the field can be expressed as [29]

$$\mathbf{p}=\frac{1}{4\omega}\mathrm{Im}\begin{bmatrix}\varepsilon\left(E_x^{*}\nabla E_x+E_y^{*}\nabla E_y+E_z^{*}\nabla E_z\right)\\+\mu\left(H_x^{*}\nabla H_x+H_y^{*}\nabla H_y+H_z^{*}\nabla H_z\right)\end{bmatrix}+\frac{1}{8\omega}\mathrm{Im}\left[\varepsilon\nabla\times\left(\mathbf{E}^{*}\times\mathbf{E}\right)+\mu\nabla\times\left(\mathbf{H}^{*}\times\mathbf{H}\right)\right]. \tag{S3}$$

This expression can be simplified introducing the 6-vector in the homogeneous medium [s2-s4]

$$\left|\Psi\right\rangle=\frac{1}{2}\begin{pmatrix}\sqrt{\varepsilon}\mathbf{E}\\ i\sqrt{\mu}\mathbf{H}\end{pmatrix}, \tag{S4}$$

resulting in

$$\mathbf{p}=\frac{1}{\hbar\omega}\left\langle\Psi\right|\hat{\mathbf{p}}_3\left(\mathbf{r}\right)\left|\Psi\right\rangle+\frac{1}{2\hbar\omega}i\hat{\mathbf{p}}_3\left(\mathbf{r}\right)\times\left\langle\Psi\right|\hat{\mathbf{S}}\left|\Psi\right\rangle, \tag{S5}$$

where $\hat{\mathbf{p}}_3(\mathbf{r})$ is the Hermitian local momentum operator in the position representation: $\hat{\mathbf{p}}_3(\mathbf{r})=\big(\delta(\hat{\mathbf{r}}-\mathbf{r})\hat{\mathbf{p}}_3+\hat{\mathbf{p}}_3\delta(\hat{\mathbf{r}}-\mathbf{r})\big)/2$ with $\hat{\mathbf{p}}_3=-i\hbar\nabla$ being the momentum operator, $\hat{\mathbf{S}}$ is the spin-1 matrix in SO(3) space [29] and $\hbar$ is the reduced Planck constant. Physically, the first and second terms in Eq. (S5) correspond to the orbital and spin parts of the momentum density, respectively. Therefore, the orbital momentum density and spin momentum density can be expressed as

$$\mathbf{p}_s = \frac{1}{8\omega}\nabla \times \mathrm{Im}\left[\varepsilon\left(\mathbf{E}^* \times \mathbf{E}\right) + \mu\left(\mathbf{H}^* \times \mathbf{H}\right)\right], \tag{S6a}$$

$$\mathbf{p}_o = \frac{1}{4\omega}\mathrm{Im}\begin{bmatrix} \varepsilon\left(E_x^*\nabla E_x + E_y^*\nabla E_y + E_z^*\nabla E_z\right) \\ +\mu\left(H_x^*\nabla H_x + H_y^*\nabla H_y + H_z^*\nabla H_z\right)\end{bmatrix}, \tag{S6b}$$

respectively. The orbital momentum density $\mathbf{p}_o = \langle\Psi|\hat{\mathbf{p}}_3(\mathbf{r})|\Psi\rangle/\hbar\omega$ is proportional to the local momentum vector. From Eq. (S6a) and the second term in Eq. (S5), the spin angular momentum (SAM) is

$$\mathbf{S} = \frac{1}{4\omega}\mathrm{Im}\left[\varepsilon\left(\mathbf{E}^* \times \mathbf{E}\right) + \mu\left(\mathbf{H}^* \times \mathbf{H}\right)\right]. \tag{S7}$$

## II. Spin-momentum relation for electromagnetic guided waves

### i. General case of the spin-momentum relationship

By employing Eq. (S6b), the *curl* of the orbital momentum density can be calculated as

$$\nabla \times \mathbf{p}_o = \frac{1}{4\omega}\mathrm{Im}\begin{bmatrix} \varepsilon\left(\nabla E_x^* \times \nabla E_x + \nabla E_y^* \times \nabla E_y + \nabla E_z^* \times \nabla E_z\right) \\ \mu\left(\nabla H_x^* \times \nabla H_x + \nabla H_y^* \times \nabla H_y + \nabla H_z^* \times \nabla H_z\right)\end{bmatrix}. \tag{S8}$$

On the other hand, from the relationship between the electric and magnetic fields within the Maxwell's theory, the curl of the spin momentum density can be evaluated as

$$\begin{aligned}\nabla \times \mathbf{p}_s &= -\frac{1}{8\omega}\nabla^2\,\mathrm{Im}\left[\varepsilon\mathbf{E}^* \times \mathbf{E} + \mu\mathbf{H}^* \times \mathbf{H}\right] \\ &= k^2\mathbf{S} - \frac{1}{4\omega}\mathrm{Im}\left\{\begin{matrix} \varepsilon\left(\frac{\partial \mathbf{E}^*}{\partial x}\times\frac{\partial \mathbf{E}}{\partial x} + \frac{\partial \mathbf{E}^*}{\partial y}\times\frac{\partial \mathbf{E}}{\partial y} + \frac{\partial \mathbf{E}^*}{\partial z}\times\frac{\partial \mathbf{E}}{\partial z}\right) \\ \mu\left(\frac{\partial \mathbf{H}^*}{\partial x}\times\frac{\partial \mathbf{H}}{\partial y} + \frac{\partial \mathbf{H}^*}{\partial y}\times\frac{\partial \mathbf{H}}{\partial y} + \frac{\partial \mathbf{H}^*}{\partial z}\times\frac{\partial \mathbf{H}}{\partial z}\right)\end{matrix}\right\},\end{aligned} \tag{S9}$$

where $k=\omega/v$ is the wave vector of the field in the medium. By introducing a Dyad's vector given by [s5]

$$\mathbf{r}_1 \otimes \mathbf{r}_2 = \begin{pmatrix} x_i^1x_i^2 & x_j^1x_i^2 & x_k^1x_i^2 \\ x_i^1x_j^2 & x_j^1x_j^2 & x_k^1x_j^2 \\ x_i^1x_k^2 & x_j^1x_k^2 & x_k^1x_k^2 \end{pmatrix}, \tag{S10}$$

where the two vectors are $\mathbf{r}_1 = \left(x_i^1, x_i^1, x_i^1\right)^{\mathrm{T}}$ and $\mathbf{r}_2 = \left(x_i^2, x_i^2, x_i^2\right)^{\mathrm{T}}$, the *curl* of the momentum density can be expressed as

$$\nabla \times \mathbf{p} = k^2\mathbf{S} - \frac{\varepsilon\mu}{4}\mathrm{Re}\left\{-\left(\nabla \otimes \mathbf{E}^*\right)\cdot\mathbf{H} - \left(\nabla \otimes \mathbf{E}\right)^{\mathrm{T}}\cdot\mathbf{H}^* + \left(\nabla \otimes \mathbf{H}^*\right)\cdot\mathbf{E} + \left(\nabla \otimes \mathbf{H}\right)^{\mathrm{T}}\cdot\mathbf{E}^*\right\}. \tag{S11}$$

Accordingly, the *curl* of the energy flow density is

$$\nabla \times \mathbf{P} = \frac{1}{\varepsilon\mu}\nabla \times \mathbf{p} = \omega^2\mathbf{S} - \frac{1}{2}\mathrm{Re}\left\{-\left(\nabla \otimes \mathbf{E}^*\right)_s\cdot\mathbf{H} + \left(\nabla \otimes \mathbf{H}^*\right)_s\cdot\mathbf{E}\right\}, \tag{S12}$$

where $(\mathbf{r}_1 \otimes \mathbf{r}_2)_s = \left\{(\mathbf{r}_1 \otimes \mathbf{r}_2) + (\mathbf{r}_1 \otimes \mathbf{r}_2)^{\mathrm{T}}\right\}/2$ [s5]. Note that the second term in the right-hand side of Eq. (S12) has a same structure as the quantum 2-form [33] that generates the Berry phase associated with a circuit, which indicates a spin-orbit interaction in the optical system (the relation between this quantum 2-form and Berry phase will be discussed in Section III).

**ii. The spin-momentum relation for the electromagnetic guided waves**

The presence of an interface between media with different relative permittivity and permeability breaks the dual symmetry between the electric and magnetic features and the intrinsic connection between the spin and energy flow densities should be considered individually for TM and TE guided modes. We first consider the situation of the guided waves on the example of a transverse magnetic (TM) surface electromagnetic wave propagating in *xy*-plane ($H_z = 0$). In this case, the evanescent field exponentially decaying in the *z*-direction can be expressed as $F(x,y)e^{-k_z z}$, where $ik_z$ is the normal to the interface component of the wave vector (Fig. 1). By employing the Maxwell's equations and the Hertz potential theory, the relation between the electric and magnetic field components can be expressed as [s6]

$$\begin{array}{ll} E_x = -\dfrac{k_z}{\beta^2}\dfrac{\partial E_z}{\partial x} & H_x = -\dfrac{i\omega\varepsilon}{\beta^2}\dfrac{\partial E_z}{\partial y} \\ E_y = -\dfrac{k_z}{\beta^2}\dfrac{\partial E_z}{\partial y} & H_y = \dfrac{i\omega\varepsilon}{\beta^2}\dfrac{\partial E_z}{\partial x} \end{array}. \tag{S13}$$

where $\beta = \sqrt{k_x^2 + k_y^2}$ represents the in-plane component of the wave vector and is related to $k_z$ by $\beta^2 + (ik_z)^2 = k^2$. Thus, for evanescent waves:

$$\begin{cases} \dfrac{\partial E_y}{\partial x} = \dfrac{\partial E_x}{\partial y} \\ H_y = -\dfrac{i\omega\varepsilon}{k_z} E_x \\ H_x = \dfrac{i\omega\varepsilon}{k_z} E_y \end{cases}. \tag{S14}$$

For TM guided modes, the 2nd term in the right-hand side of Eq. (S12) can be expressed as

$$\begin{aligned} &\mathrm{Re}\left\{-(\nabla \otimes \mathbf{E}^*)_s \cdot \mathbf{H} + (\nabla \otimes \mathbf{H}^*)_s \cdot \mathbf{E}\right\} \\ &= \frac{1}{2}\mathrm{Re}\begin{pmatrix} \dfrac{\partial H_x^*}{\partial x}E_x + \dfrac{\partial H_y^*}{\partial x}E_y + \dfrac{\partial H_x}{\partial x}E_x^* + \dfrac{\partial H_x}{\partial y}E_y^* + \dfrac{\partial H_x}{\partial z}E_z^* - \dfrac{\partial E_x^*}{\partial x}H_x - \dfrac{\partial E_y^*}{\partial x}H_y - \dfrac{\partial E_x}{\partial x}H_x^* - \dfrac{\partial E_x}{\partial y}H_y^* \\ \dfrac{\partial H_x^*}{\partial y}E_x + \dfrac{\partial H_y^*}{\partial y}E_y + \dfrac{\partial H_y}{\partial x}E_x^* + \dfrac{\partial H_y}{\partial y}E_y^* + \dfrac{\partial H_y}{\partial z}E_z^* - \dfrac{\partial E_x^*}{\partial y}H_x - \dfrac{\partial E_y^*}{\partial y}H_y - \dfrac{\partial E_y}{\partial x}H_x^* - \dfrac{\partial E_y}{\partial y}H_y^* \\ \dfrac{\partial H_x^*}{\partial z}E_x + \dfrac{\partial H_y^*}{\partial z}E_y - \dfrac{\partial E_x^*}{\partial z}H_x - \dfrac{\partial E_y^*}{\partial z}H_y - \dfrac{\partial E_z}{\partial x}H_x^* - \dfrac{\partial E_z}{\partial y}H_y^* \end{pmatrix} \end{aligned} \tag{S15}$$

and, by employing the relations from Eq. (S14), it can be rewritten as

$$\mathrm{Re}\left\{-\left(\nabla\otimes\mathbf{E}^*\right)_s\cdot\mathbf{H}+\left(\nabla\otimes\mathbf{H}^*\right)_s\cdot\mathbf{E}\right\}=\frac{1}{2}\mathrm{Re}\begin{pmatrix} i\omega\varepsilon E_z E_y^* - i\omega\varepsilon E_y E_z^* \\ -i\omega\varepsilon E_x^* E_z + i\omega\varepsilon E_x E_z^* \\ \frac{i\omega\varepsilon}{k_z}\frac{\partial E_z}{\partial x}E_y^* - \frac{i\omega\varepsilon}{k_z}\frac{\partial E_z}{\partial y}E_x^* \end{pmatrix}. \tag{S16}$$

Taking into account the connotative relations from Eq. (S13):

$$\begin{cases} \frac{\partial E_z}{\partial y} = i\omega\mu H_x - k_z E_y \\ \frac{\partial E_z}{\partial x} = -k_z E_x - i\omega\mu H_y \end{cases}, \tag{S17}$$

Eq. (S16) can further be written as

$$\mathrm{Re}\left\{-\left(\nabla\otimes\mathbf{E}^*\right)_s\cdot\mathbf{H}+\left(\nabla\otimes\mathbf{H}^*\right)_s\cdot\mathbf{E}\right\}=\frac{1}{2}\mathrm{Re}\begin{pmatrix} i\omega\varepsilon E_z E_y^* - i\omega\varepsilon E_y E_z^* \\ -i\omega\varepsilon E_x^* E_z + i\omega\varepsilon E_x E_z^* \\ \left(-i\omega\varepsilon E_x E_y^* + \frac{\omega^2\varepsilon\mu}{k_z}H_y E_y^*\right) - \left(-\frac{\omega^2\varepsilon\mu}{k_z}H_x E_x^* - i\omega\varepsilon E_y E_x^*\right) \end{pmatrix}. \tag{S18}$$

Finally, by employing the last two relations in Eq. (S13), the above equation can be presented as

$$\mathrm{Re}\left\{-\left(\nabla\otimes\mathbf{E}^*\right)_s\cdot\mathbf{H}+\left(\nabla\otimes\mathbf{H}^*\right)_s\cdot\mathbf{E}\right\}=\frac{1}{2}\mathrm{Re}\begin{pmatrix} i\omega\varepsilon\left(E_y^* E_z - E_y E_z^*\right) \\ i\omega\varepsilon\left(E_z^* E_x - E_z E_x^*\right) \\ i\omega\varepsilon\left(E_x^* E_y - E_x E_y^*\right) + i\omega\mu\left(H_x^* H_y - H_x H_y^*\right) \end{pmatrix}. \tag{S19}$$

As a result, the *curl* of the energy flow density can be expressed as

$$\nabla\times\mathbf{P} = \omega^2\mathbf{S} - \frac{1}{4}\mathrm{Re}\begin{pmatrix} i\omega\varepsilon\left(E_y^* E_z - E_y E_z^*\right) \\ i\omega\varepsilon\left(E_z^* E_x - E_z E_x^*\right) \\ i\omega\varepsilon\left(E_x^* E_y - E_x E_y^*\right) + i\omega\mu\left(H_x^* H_y - H_x H_y^*\right) \end{pmatrix} = 2\omega^2\mathbf{S}. \tag{S20}$$

Similarly, for the TE evanescent wave ($E_z = 0$), e.g., Bloch surface wave [s7], the field components fulfill the following conditions [s6]:

$$\begin{aligned} E_x &= \frac{i\omega\mu}{\beta^2}\frac{\partial H_z}{\partial y} & H_x &= -\frac{k_z}{\beta^2}\frac{\partial H_z}{\partial x} \\ E_y &= -\frac{i\omega\mu}{\beta^2}\frac{\partial H_z}{\partial x} & H_y &= -\frac{k_z}{\beta^2}\frac{\partial H_z}{\partial y} \end{aligned} \tag{S21}$$

and the curl of the energy flow density also is

$$\nabla\times\mathbf{P} = \omega^2\mathbf{S} - \frac{1}{4}\mathrm{Re}\left\{i\omega\begin{pmatrix} \mu\left(H_y^* H_z - H_z^* H_y\right) \\ \mu\left(H_z^* H_x - H_x^* H_z\right) \\ \mu\left(H_x^* H_y - H_y^* H_x\right) + \varepsilon\left(E_x^* E_y - E_y^* E_x\right) \end{pmatrix}\right\} = 2\omega^2\mathbf{S}. \tag{S22}$$

As the result, for both the TM and TE guided modes, the spin angular momentum and energy flow density (or momentum density) fulfill the relationship

$$\mathbf{S} = \frac{1}{2\omega^2}\nabla\times\mathbf{P} = \frac{1}{2k^2}\nabla\times\mathbf{p}\,. \tag{S23}$$

It worth noting that the orbital angular momentum of optical vortex results from the rotation of photon momentum (**L** = **r**×**P**$_o$, where **r** is the position vector and **P**$_o$ is the orbital flow density). It is associated to the global phase structure of a beam. Whereas in our case, the transverse optical spin of an electromagnetic field originates from the curl/vorticity of energy flow density (**P**). It is associated to the 'local' rotation of polarization states in the photon transportation.

### III. Boundary conditions for the spin-momentum equations

In order to derive the boundary conditions for SAM (**S**) and energy flow density (**P**) /momentum density (**p**) at an optical interface supporting guided modes, we consider a guided wave confined at an interface between medium 1 and medium 2 (Fig. S1).

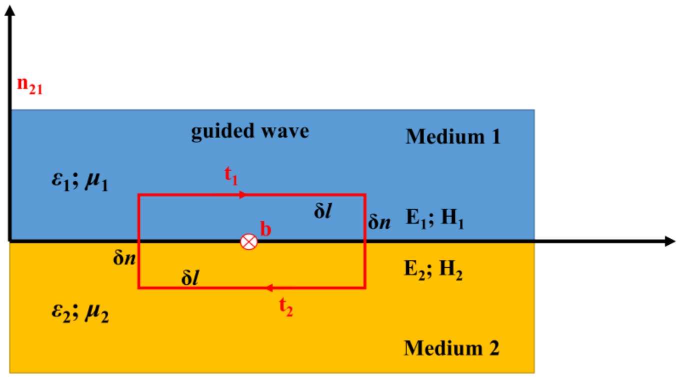


**Fig. S1. Schematic diagram of an optical interface supporting guided modes used for the derivation of the boundary conditions for SAM S and momentum density p.** $\varepsilon_i$ and $\mu_i$ (i=1, 2) are the permittivity and permeability of the media in the upper and lower space, $E_i$ and $H_i$ are the electric and magnetic field, ω is the angular frequency of the guide wave, and $n_{21}$ is the unit normal vector from medium 2 to medium 1.

#### i. Boundary condition for the out-of-plane component of Poynting vector and momentum density

For a monochromatic, time-harmonic electromagnetic wave propagating in generally inhomogeneous, medium, the energy flow density is divergence-free, i.e.,

$$\nabla\cdot\mathbf{P} = \nabla\cdot\frac{1}{2}\mathrm{Re}\left\{\mathbf{E}^*\times\mathbf{H}\right\} = \frac{1}{2}\mathrm{Re}\left\{\mathbf{H}\cdot\left(\nabla\times\mathbf{E}^*\right)-\mathbf{E}^*\cdot\left(\nabla\times\mathbf{H}\right)\right\} = \frac{1}{2}\mathrm{Re}\left\{i\omega\left(\varepsilon\mathbf{E}^*\cdot\mathbf{E}-\mu\mathbf{H}\cdot\mathbf{H}^*\right)\right\} = 0\,.$$

Here, **E** and **H** stand for the electric and magnetic field in an arbitrary inhomogeneous medium, ε and μ are the permittivity and permeability of the inhomogeneous medium, the superscript * represents the complex conjugate. Thus, the out-of-plane energy flow density is continuous at an interface

$$\mathbf{n}_{21}\cdot\left(\mathbf{P}_1-\mathbf{P}_2\right)=0\,. \tag{S24}$$

On the other hand, the momentum density is related to the Poynting vector by **p**=*εμ***P**. Thus, the divergence of momentum density is

$$\nabla\cdot\mathbf{p}=\nabla\cdot\frac{\varepsilon\mu}{2}\mathrm{Re}\left\{\mathbf{E}^{*}\times\mathbf{H}\right\}=\frac{\varepsilon\mu}{2}\nabla\cdot\mathrm{Re}\left\{\mathbf{E}^{*}\times\mathbf{H}\right\}+\frac{\nabla(\mu\varepsilon)}{2}\cdot\mathrm{Re}\left\{\mathbf{E}^{*}\times\mathbf{H}\right\}. \qquad \text{(S25)}$$

The first term in the right side of Eq. (S25) vanishes because of the divergence-free of Poynting vector. To obtain the boundary condition, we apply the Gauss's theorem to Eq. (S25):

$$\oiint_{\sigma}\mathbf{p}d\sigma=\iiint_{V}\frac{\nabla(\mu\varepsilon)}{2}\cdot\mathrm{Re}\left\{\mathbf{E}^{*}\times\mathbf{H}\right\}dV,$$

from which one can obtain

$$\mathbf{p}_1\cdot\mathbf{n}_1\delta A+\mathbf{p}_2\cdot\mathbf{n}_2\delta A=\frac{\partial(\mu\varepsilon)}{\partial n}\mathbf{n}_{21}\cdot\frac{1}{2}\mathrm{Re}\left\{\mathbf{E}^{*}\times\mathbf{H}\right\}\delta A\delta n. \qquad \text{(S26)}$$

Here, $\mathbf{n}_{21}\cdot\mathrm{Re}\left\{\mathbf{E}^{*}\times\mathbf{H}\right\}$, which is related to the in-plane electric and magnetic field components, are continuous through the interface. Thus, Eq. (S26) can be re-written as

$$\mathbf{n}_{21}\cdot(\mathbf{p}_1-\mathbf{p}_2)=(\mu_1\varepsilon_1-\mu_2\varepsilon_2)\mathbf{n}_{21}\cdot\frac{1}{2}\mathrm{Re}\left\{\mathbf{E}_1^{*}\times\mathbf{H}_1\right\}=(\mu_1\varepsilon_1-\mu_2\varepsilon_2)\mathbf{n}_{21}\cdot\frac{1}{2}\mathrm{Re}\left\{\mathbf{E}_2^{*}\times\mathbf{H}_2\right\}. \qquad \text{(S27)}$$

This equation indicates that the out-of-plane momentum density is not continuous at an interface due to the discontinuity of the permittivity and permeability.

**ii. Boundary condition for the in-plane component of Poynting vector and momentum density**

In a general case, the difference of the in-plane energy flow density right above and below an interface can be expressed as

$$(\mathbf{P}_1\cdot\mathbf{t}_1-\mathbf{P}_2\cdot\mathbf{t}_2)=\frac{1}{2}\mathrm{Re}\left\{\begin{matrix}\left[E_b^{*}\left(\frac{1}{\mu_1}B_{1n}-\frac{1}{\mu_2}B_{2n}\right)-\left(\frac{1}{\varepsilon_1}D_{1n}^{*}-\frac{1}{\varepsilon_2}D_{2n}^{*}\right)H_b\right]\mathbf{t}\\ \left[\left(\frac{1}{\varepsilon_1}D_{1n}^{*}-\frac{1}{\varepsilon_2}D_{2n}^{*}\right)H_t-E_t^{*}\left(\frac{1}{\mu_1}B_{1n}-\frac{1}{\mu_2}B_{2n}\right)\right]\mathbf{b}\end{matrix}\right\}.$$

Note that the physical quantities: $E_b$, $E_t$, $D_n$, $H_b$, $H_t$, $B_n$ are continuous at the interface. For a transverse magnetic (TM) mode, which has $H_n = 0$,

$$(\mathbf{P}_1\cdot\mathbf{t}_1-\mathbf{P}_2\cdot\mathbf{t}_2)=\frac{1}{2}\mathrm{Re}\left\{\begin{matrix}\left[\left(\mathbf{n}_{21}\cdot\nabla\frac{1}{\mu}\right)E_b^{*}B_n-\left(\mathbf{n}_{21}\cdot\nabla\frac{1}{\varepsilon}\right)D_n^{*}H_b\right]\mathbf{t}\\ \left[\left(\mathbf{n}_{21}\cdot\nabla\frac{1}{\varepsilon}\right)D_n^{*}H_t-\left(\mathbf{n}_{21}\cdot\nabla\frac{1}{\mu}\right)E_t^{*}B_n\right]\mathbf{b}\end{matrix}\right\}=\left(\mathbf{n}_{21}\cdot\nabla\frac{1}{\varepsilon}\right)\frac{1}{2}\mathrm{Re}\left\{\begin{matrix}-D_n^{*}H_b\mathbf{t}\\ D_n^{*}H_t\mathbf{b}\end{matrix}\right\}\delta n$$

$$=\left(\frac{1}{\varepsilon_1}-\frac{1}{\varepsilon_2}\right)\varepsilon_1\mathbf{P}_{1t}=\left(\frac{1}{\varepsilon_1}-\frac{1}{\varepsilon_2}\right)\varepsilon_2\mathbf{P}_{2t}. \qquad \text{(S28)}$$

While for a transverse electric (TE) mode, which has $E_n = 0$,

$$\left(\mathbf{P}_1\cdot\mathbf{t}_1-\mathbf{P}_2\cdot\mathbf{t}_2\right)=\frac{1}{2}\mathrm{Re}\left\{\begin{matrix}\left[\left(\mathbf{n}_{21}\cdot\nabla\frac{1}{\mu}\right)E_b^*B_n-\left(\mathbf{n}_{21}\cdot\nabla\frac{1}{\varepsilon}\right)D_n^*H_b\right]\mathbf{t}\\ \left[\left(\mathbf{n}_{21}\cdot\nabla\frac{1}{\varepsilon}\right)D_n^*H_t-\left(\mathbf{n}_{21}\cdot\nabla\frac{1}{\mu}\right)E_t^*B_n\right]\mathbf{b}\end{matrix}\right\}=\left(\mathbf{n}_{21}\cdot\nabla\frac{1}{\mu}\right)\frac{1}{2}\mathrm{Re}\left\{\begin{matrix}E_b^*B_n\mathbf{t}\\ -E_t^*B_n\mathbf{b}\end{matrix}\right\}\delta n$$

$$=\left(\frac{1}{\mu_1}-\frac{1}{\mu_2}\right)\mu_1\mathbf{P}_{1t}=\left(\frac{1}{\mu_1}-\frac{1}{\mu_2}\right)\mu_2\mathbf{P}_{2t}\,. \tag{S29}$$

Here, the unit vector **t** is along the $\mathbf{t}_1$ direction (Fig. S1). Eqs. (S28) and (S29) can be rewritten as

$$\begin{matrix}\mathbf{n}_{21}\times\left(\varepsilon_1\mathbf{P}_1-\varepsilon_2\mathbf{P}_2\right)=0 & \text{for TM mode}\\ \mathbf{n}_{21}\times\left(\mu_1\mathbf{P}_1-\mu_2\mathbf{P}_2\right)=0 & \text{for TE mode}\end{matrix}\,. \tag{S30}$$

On the other hand, the difference of the in-plane momentum density right above and below an interface can be expressed as

$$\left(\mathbf{p}_1\cdot\mathbf{t}_1-\mathbf{p}_2\cdot\mathbf{t}_2\right)=\frac{1}{2}\mathrm{Re}\left\{\begin{matrix}\left[\left(\varepsilon_1-\varepsilon_2\right)E_b^*B_n-\left(\mu_1-\mu_2\right)D_n^*H_b\right]\mathbf{t}\\ \left[\left(\mu_1-\mu_2\right)D_n^*H_t-\left(\varepsilon_1-\varepsilon_2\right)E_t^*B_n\right]\mathbf{b}\end{matrix}\right\}.$$

For a transverse magnetic (TM) mode, which has $H_n = 0$,

$$\left(\mathbf{p}_1\cdot\mathbf{t}_1-\mathbf{p}_2\cdot\mathbf{t}_2\right)=\frac{\mathbf{n}_{21}\cdot\nabla\mu}{2}\mathrm{Re}\left\{\begin{matrix}\left[-D_n^*H_b\right]\mathbf{t}\\ \left[D_n^*H_t\right]\mathbf{b}\end{matrix}\right\}\delta n=\left(\mu_1-\mu_2\right)\frac{1}{\mu_1}\mathbf{p}_{1t}=\left(\mu_1-\mu_2\right)\frac{1}{\mu_2}\mathbf{p}_{2t}\,. \tag{S31}$$

While for a transverse electric (TE) mode, which has $E_n = 0$,

$$\left(\mathbf{p}_1\cdot\mathbf{t}_1-\mathbf{p}_2\cdot\mathbf{t}_2\right)=\frac{\mathbf{n}_{21}\cdot\nabla\varepsilon}{2}\mathrm{Re}\left\{\begin{matrix}\left[E_b^*B_n\right]\mathbf{t}\\ \left[-E_t^*B_n\right]\mathbf{b}\end{matrix}\right\}\delta n=\left(\varepsilon_1-\varepsilon_2\right)\frac{1}{\varepsilon_1}\mathbf{p}_{1t}=\left(\varepsilon_1-\varepsilon_2\right)\frac{1}{\varepsilon_2}\mathbf{p}_{2t}\,. \tag{S32}$$

Eqs. (S31) and (S32) can be rewritten as

$$\begin{matrix}\mathbf{n}_{21}\times\left(\frac{1}{\mu_1}\mathbf{p}_1-\frac{1}{\mu_2}\mathbf{p}_2\right)=0 & \text{for TM mode}\\ \mathbf{n}_{21}\times\left(\frac{1}{\varepsilon_1}\mathbf{p}_1-\frac{1}{\varepsilon_1}\mathbf{p}_2\right)=0 & \text{for TE mode}\end{matrix}\,. \tag{S33}$$

**iii. Boundary condition for the out-of-plane component of spin angular momentum**

The divergence of spin angular momentum in generally inhomogeneous medium can be expressed as

$$\nabla\cdot\mathbf{S}=\nabla\cdot\frac{1}{4\omega}\mathrm{Im}\left\{\varepsilon\left(\mathbf{E}^*\times\mathbf{E}\right)+\mu\left(\mathbf{H}^*\times\mathbf{H}\right)\right\}$$

$$=\frac{1}{4\omega}\mathrm{Im}\left\{\nabla\varepsilon\cdot\left(\mathbf{E}^*\times\mathbf{E}\right)+\varepsilon\nabla\cdot\left(\mathbf{E}^*\times\mathbf{E}\right)+\nabla\mu\cdot\left(\mathbf{H}^*\times\mathbf{H}\right)+\mu\nabla\cdot\left(\mathbf{H}^*\times\mathbf{H}\right)\right\}$$

$$= \frac{1}{4\omega}\text{Im}\left\{\nabla\varepsilon\cdot\left(\mathbf{E}^*\times\mathbf{E}\right)+\nabla\mu\cdot\left(\mathbf{H}^*\times\mathbf{H}\right)\right\}. \tag{S34}$$

At an interface between two homogeneous media (Fig. S1), the permittivity and permeability undergo an abrupt change along the normal direction and hence $\nabla\varepsilon=\mathbf{n}_{21}\partial\varepsilon/\partial n$ and $\nabla\mu=\mathbf{n}_{21}\partial\mu/\partial n$. To obtain the boundary condition, we apply the Gauss's theorem to Eq. (S34):

$$\oiint_\sigma \mathbf{S}d\sigma=\iiint_V \frac{1}{4\omega}\text{Im}\left\{\nabla\varepsilon\cdot\left(\mathbf{E}^*\times\mathbf{E}\right)+\nabla\mu\cdot\left(\mathbf{H}^*\times\mathbf{H}\right)\right\}dV,$$

from which one can obtain

$$\mathbf{S}_1\cdot\mathbf{n}_1\delta A+\mathbf{S}_2\cdot\mathbf{n}_2\delta A=\frac{1}{4\omega}\text{Im}\left\{\frac{\partial\varepsilon}{\partial n}\mathbf{n}_{21}\cdot\left(\mathbf{E}^*\times\mathbf{E}\right)+\frac{\partial\mu}{\partial n}\mathbf{n}_{21}\cdot\left(\mathbf{H}^*\times\mathbf{H}\right)\right\}\delta A\delta n. \tag{S35}$$

Here, $\mathbf{n}_{21}\cdot\left(\mathbf{E}^*\times\mathbf{E}\right)$ and $\mathbf{n}_{21}\cdot\left(\mathbf{H}^*\times\mathbf{H}\right)$, which are related to the in-plane electric and magnetic field components, are continuous through the interface. Thus, Eq. (S35) can be re-written as

$$\mathbf{n}_{21}\cdot\left(\mathbf{S}_1-\mathbf{S}_2\right)=\frac{1}{4\omega}\text{Im}\left\{\left(\varepsilon_1-\varepsilon_2\right)\mathbf{n}_{21}\cdot\left(\mathbf{E}_1^*\times\mathbf{E}_1\right)+\left(\mu_1-\mu_2\right)\mathbf{n}_{21}\cdot\left(\mathbf{H}_1^*\times\mathbf{H}_1\right)\right\}$$

$$=\frac{1}{4\omega}\text{Im}\left\{\left(\varepsilon_1-\varepsilon_2\right)\mathbf{n}_{21}\cdot\left(\mathbf{E}_2^*\times\mathbf{E}_2\right)+\left(\mu_1-\mu_2\right)\mathbf{n}_{21}\cdot\left(\mathbf{H}_2^*\times\mathbf{H}_2\right)\right\}. \tag{S36}$$

This equation indicates that the out-of-plane spin component is not continuous at an interface due to the discontinuity of the permittivity and permeability.

**vi. Boundary condition for the in-plane component of spin angular momentum**

The curl of spin angular momentum is

$$\nabla\times\mathbf{S}=\nabla\times\frac{1}{4\omega}\text{Im}\left\{\varepsilon\left(\mathbf{E}^*\times\mathbf{E}\right)+\mu\left(\mathbf{H}^*\times\mathbf{H}\right)\right\}$$

$$=\frac{1}{4\omega}\text{Im}\left\{\nabla\varepsilon\times\left(\mathbf{E}^*\times\mathbf{E}\right)+\nabla\mu\times\left(\mathbf{H}^*\times\mathbf{H}\right)\right\}+\frac{\varepsilon\mu}{4\omega}\text{Im}\left\{\frac{1}{\mu}\nabla\times\left(\mathbf{E}^*\times\mathbf{E}\right)+\frac{1}{\varepsilon}\nabla\times\left(\mathbf{H}^*\times\mathbf{H}\right)\right\}$$

$$=\frac{1}{4\omega}\text{Im}\left\{\nabla\varepsilon\times\left(\mathbf{E}^*\times\mathbf{E}\right)+\nabla\mu\times\left(\mathbf{H}^*\times\mathbf{H}\right)\right\}+2\varepsilon\mu\mathbf{P}_s. \tag{S37}$$

To obtain the boundary condition, we apply the Stokes's theorem to Eq. (S37):

$$\oint_l \mathbf{S}dl=\iint_\sigma\left[\frac{1}{4\omega}\text{Im}\left\{\nabla\varepsilon\times\left(\mathbf{E}^*\times\mathbf{E}\right)+\nabla\mu\times\left(\mathbf{H}^*\times\mathbf{H}\right)\right\}+2\varepsilon\mu\mathbf{P}_s\right]d\sigma,$$

from which one can obtain:

$$\left(\mathbf{S}_1\cdot\mathbf{t}_1\delta l+\mathbf{S}_2\cdot\mathbf{t}_2\delta l\right)=\left[\frac{1}{4\omega}\text{Im}\left\{\frac{\partial\varepsilon}{\partial n}\mathbf{n}_{21}\times\left(\mathbf{E}^*\times\mathbf{E}\right)+\frac{\partial\mu}{\partial n}\mathbf{n}_{21}\times\left(\mathbf{H}^*\times\mathbf{H}\right)\right\}+2\varepsilon\mu\mathbf{P}_s\right]\cdot\mathbf{b}\delta l\delta n.$$

Here, **b=n×t** is the unit vector normal to the **n-t** plane (Fig. S1). The condition $\delta n \to 0$ leads to the vanishing of the last term: $2\varepsilon\mu\mathbf{P}_s\delta l\delta n$ . On the other hand, although $\mathbf{n}_{21}\times\left(\mathbf{E}^*\times\mathbf{E}\right)$ and $\mathbf{n}_{21}\times\left(\mathbf{H}^*\times\mathbf{H}\right)$, which are related to the in-plane spin component, are not continuous because of the discontinuity of the out-of-plane electric/magnetic field component, $\varepsilon\mathbf{n}_{21}\times\left(\mathbf{E}^*\times\mathbf{E}\right)$ and $\mu\mathbf{n}_{21}\times\left(\mathbf{H}^*\times\mathbf{H}\right)$ are, nevertheless, continuous since $D_n$ ($=\varepsilon E_n$) and $B_n$ ($=\mu H_n$) are continuous in a source-free system, where $D_n$ and $B_n$ are the normal-to-the-interface component of electric displacement and magnetic flux density. As the result, the boundary condition is

$$\mathbf{n}_{21}\times\left(\mathbf{S}_1-\mathbf{S}_2\right)=0\,, \tag{S38}$$

which indicates that the in-plane spin angular momentum is continuous at an interface.

To summarize, we have obtained the boundary conditions for the Poynting vector (Eqs. (S24) and (S30)), momentum density (Eqs. (S27) and (S33)) and SAM (Eq. (S36) and (S38)) for an arbitrary guided wave an interface between two media.

**v. P, p and S boundary condition for surface plasmon polaritons**

For SPPs, which are TM modes propagating along a metal-dielectric interface (with $\mu_1=\mu_2$), the boundary condition for the out-of-plane spin angular momentum (Eq. (S36) becomes

$$\mathbf{n}_{21}\cdot\left(\mathbf{S}_1-\mathbf{S}_2\right)=\frac{1}{4\omega}\mathrm{Im}\left\{\left(\varepsilon_1-\varepsilon_2\right)\mathbf{n}_{21}\cdot\left(\mathbf{E}_1^*\times\mathbf{E}_1\right)\right\}. \tag{S39}$$

From the relationship between the field components given by Eq. (S13), the magnetic contribution to the out-of-plane spin angular momentum is proportional to the electric contribution and one can rewrite the out-of-plane spin angular momentum as:

$$\mathbf{n}_{21}\cdot\mathbf{S}=\mathbf{n}_{21}\cdot\frac{1}{4\omega}\mathrm{Im}\left\{\varepsilon\left(\mathbf{E}^*\times\mathbf{E}\right)+\mu\left(\mathbf{H}^*\times\mathbf{H}\right)\right\}=\frac{1}{4\omega}\mathrm{Im}\left\{\varepsilon\frac{\beta^2}{k_n^2}\mathbf{n}_{21}\cdot\left(\mathbf{E}^*\times\mathbf{E}\right)\right\}, \tag{S40}$$

where $\beta$ and $ik_n$ stand for the in-plane and out-of-plane wave-vector component of SPP, respectively. Substituting Eq. (S40) into Eq. (S39) and taking into account that in the upper half space (dielectric space) $\beta^2/k_{n,1}^2=-\varepsilon_2/\varepsilon_1$ (from the SPP dispersion relation), one can obtain

$$\mathbf{n}_{21}\cdot\left(\mathbf{S}_1-\mathbf{S}_2\right)=-\frac{\varepsilon_1-\varepsilon_2}{\varepsilon_2}\mathbf{n}_{21}\cdot\mathbf{S}_1$$

and finally:

$$\mathbf{n}_{21}\cdot\left(\varepsilon_1\mathbf{S}_1-\varepsilon_2\mathbf{S}_2\right)=0\,.$$

In addition, for SPPs, the momentum density, which is parallel to the energy flow density, has only the in-plane component. Thus, the out-of-plane momentum density is continuous through the interface according to Eq. (S27).

As a result, the boundary conditions for **P**, **p** and **S** for SPP waves can be summarized as

$$\begin{cases} \mathbf{n}_{21}\cdot(\mathbf{P}_1-\mathbf{P}_2)=0,\ \mathbf{n}_{21}\cdot(\mathbf{p}_1-\mathbf{p}_2)=0 \\ \mathbf{n}_{21}\times(\varepsilon_1\mathbf{P}_1-\varepsilon_2\mathbf{P}_2)=0,\ \ \mathbf{n}_{21}\times(\mathbf{p}_1-\mathbf{p}_2)=0 \\ \mathbf{n}_{21}\cdot(\varepsilon_1\mathbf{S}_1-\varepsilon_2\mathbf{S}_2)=0 \\ \mathbf{n}_{21}\times(\mathbf{S}_1-\mathbf{S}_2)=0 \end{cases}. \tag{S41}$$

The boundary conditions (Eq. (S41)) clearly indicate the flip of the out-of-plane spin and the in-plane Poynting vector of SPP across the metal/dielectric interface due to the opposite signs of the permittivities; while the in-plane momentum density and SAM are continuous across the interface. The in-plane Poynting vector is reversed across the interface because of the opposite signs of the permittivities, while the in-plane momentum density is continuous as the momentum density is related to Poynting vector via *ε*. Also for the curl-relationship, the spin is related to the Poynting vector via *ω* and so the spin-momentum locking property is consistent in the dielectric and metal (right-handed rule). At the same time, the spin is related to the momentum density via *k*, which is related to *ε*, so that the spin-momentum locking obeys the right-handed rule in the dielectric but the left-handed rule in the metal.

## IV. Discussion on the spin-orbit interaction and spin topological properties for electromagnetic guided waves

### i. Optical Dirac equation and spin-orbit interaction

The Dirac equation is originally derived for a spin-1/2 particle [s8]:

$$i\hbar\frac{\partial}{\partial t}\Phi=\hat{\mathbf{H}}\Phi=\left(c\boldsymbol{\alpha}\cdot\mathbf{p}+\boldsymbol{\beta}mc^2\right)\Phi\ . \tag{S42}$$

Here, $\hat{\mathbf{H}}$ denotes the Hamiltonian operator, Φ is the electric wave function and the four Dirac matrices can be expressed in terms of the Pauli matrices:

$$\begin{aligned} \boldsymbol{\alpha}_i&=\begin{pmatrix}0&\sigma_i\\ \sigma_i&0\end{pmatrix}=\sigma_x\odot\sigma_i \\ \boldsymbol{\beta}&=\begin{pmatrix}\sigma_0&0\\ 0&-\sigma_0\end{pmatrix}=\sigma_z\odot\sigma_0 \end{aligned}, \tag{S43}$$

where *i* = *x, y, z* and the Pauli matrices are

$$\sigma_0=\begin{pmatrix}1&0\\0&1\end{pmatrix}\quad \sigma_x=\begin{pmatrix}0&1\\1&0\end{pmatrix}\quad \sigma_y=\begin{pmatrix}0&-i\\i&0\end{pmatrix}\quad \sigma_z=\begin{pmatrix}1&0\\0&-1\end{pmatrix}.$$

For the time-harmonic electromagnetic field propagating in the homogeneous medium, where is in the absence of charges and currents, the Maxwell's equation can be written as

$$\begin{cases} \nabla\cdot\mathbf{E}=0 \\ \nabla\cdot(\mu\mathbf{H})=\nabla\cdot(\mu_0\mu_r\mathbf{H})=0 \\ \nabla\times\mathbf{E}=-\mu\frac{\partial\mathbf{H}}{\partial t}=-\mu_0\frac{\partial\mathbf{H}}{\partial t}-\mu_0(\mu_r-1)\frac{\partial\mathbf{H}}{\partial t} \\ \nabla\times\mathbf{H}=\varepsilon\frac{\partial\mathbf{E}}{\partial t}=\varepsilon_0\frac{\partial\mathbf{E}}{\partial t}+\varepsilon_0(\varepsilon_r-1)\frac{\partial\mathbf{E}}{\partial t} \end{cases}, \tag{S44}$$

where $\varepsilon_r$ and $\mu_r$ are the relative permittivity and permeability, respectively. Here, with the identity for arbitrary two vectors **A** and **B**: $\mathbf{A}\times\mathbf{B}=-i\left(\mathbf{A}\cdot\hat{\mathbf{S}}\right)\mathbf{B}$ [29], where $\hat{\mathbf{S}}$ is the spin-1 matrix in SO(3) expressed as:

$$\hat{\mathbf{S}}=\left\{\hat{S}_x,\hat{S}_y,\hat{S}_z\right\}=\left\{\begin{pmatrix}0&0&0\\0&0&i\\0&-i&0\end{pmatrix},\begin{pmatrix}0&0&-i\\0&0&0\\i&0&0\end{pmatrix},\begin{pmatrix}0&i&0\\-i&0&0\\0&0&0\end{pmatrix}\right\}, \tag{S45}$$

the *curl* operator can be rewritten as

$$\nabla\times=-i\left(\hat{\mathbf{S}}\cdot\nabla\right)=\frac{1}{\hbar}\hat{\mathbf{S}}\cdot\hat{\mathbf{p}}. \tag{S46}$$

Firstly, if we consider the electromagnetic field in the homogeneous space, the latter two equations of Eq. (S44) can be written as

$$\hat{\mathbf{H}}\left|\Psi\right\rangle=v\begin{pmatrix}\mathbf{0}&\hat{\mathbf{S}}\\\hat{\mathbf{S}}&\mathbf{0}\end{pmatrix}\cdot\hat{\mathbf{p}}\left|\Psi\right\rangle=i\hbar\frac{\partial}{\partial t}\left|\Psi\right\rangle. \tag{S47}$$

The Hamiltonian operator $\hat{\mathbf{H}}$ is

$$\hat{\mathbf{H}}=v\hat{\boldsymbol{\tau}}\cdot\hat{\mathbf{p}}=v\begin{pmatrix}\mathbf{0}&\hat{\mathbf{S}}\\\hat{\mathbf{S}}&\mathbf{0}\end{pmatrix}\cdot\hat{\mathbf{p}}, \tag{S48}$$

where $v\hat{\boldsymbol{\tau}}$ denotes the energy flow density operator (the momentum density operator is $\hat{\boldsymbol{\tau}}/v$ ) and the corresponding energy flow density can be expressed as

$$\mathbf{P}=v^2\mathbf{p}=\frac{1}{2}\mathrm{Re}\left\{\mathbf{E}^*\times\mathbf{H}\right\}=\left\langle\Psi\right|v\begin{pmatrix}\mathbf{0}&\hat{\mathbf{S}}\\\hat{\mathbf{S}}&\mathbf{0}\end{pmatrix}\left|\Psi\right\rangle=\left\langle\Psi\right|v\hat{\boldsymbol{\tau}}\left|\Psi\right\rangle. \tag{S49}$$

Interestingly, the 1st-order partial derivative of position operator is

$$\dot{\mathbf{r}}=\frac{i}{\hbar}\left[\hat{\mathbf{H}},\mathbf{r}\right]=v\begin{pmatrix}0&\hat{\mathbf{S}}\\\hat{\mathbf{S}}&0\end{pmatrix}=v\hat{\boldsymbol{\tau}}, \tag{S50}$$

which indicates energy flow density operator has the property of a velocity operator and can describe the photon trajectory in accord with the momentum operator. Moreover, the SAM operator of electromagnetic wave is

$$\hat{\boldsymbol{\Sigma}}=\hbar\begin{bmatrix}\hat{\mathbf{S}}&0\\0&\hat{\mathbf{S}}\end{bmatrix}, \tag{S51}$$

and the corresponding SAM can be expressed as

$$\mathbf{S}=\frac{1}{4\omega}\mathrm{Im}\left\{\varepsilon\mathbf{E}^*\times\mathbf{E}+\mu\mathbf{H}^*\times\mathbf{H}\right\}=\frac{1}{\hbar\omega}\left\langle\Psi\right|\hbar\begin{bmatrix}\hat{\mathbf{S}}&0\\0&\hat{\mathbf{S}}\end{bmatrix}\left|\Psi\right\rangle=\frac{1}{\hbar\omega}\left\langle\Psi\right|\hat{\boldsymbol{\Sigma}}\left|\Psi\right\rangle. \tag{S52}$$

Thus, we can calculate that

$$\dot{\hat{\boldsymbol{\Sigma}}} = \frac{i}{\hbar}\left[\hat{\mathbf{H}}, \hat{\boldsymbol{\Sigma}}\right] = -v\hat{\boldsymbol{\tau}} \times \hat{\mathbf{p}} \text{ and } \dot{\hat{\mathbf{L}}} = \frac{i}{\hbar}\left[\hat{\mathbf{H}}, \hat{\mathbf{L}}\right] = v\hat{\boldsymbol{\tau}} \times \hat{\mathbf{p}} , \tag{S53}$$

where the OAM operator is $\hat{\mathbf{L}} = \hat{\mathbf{r}} \times \hat{\mathbf{p}}$. These equations show that the SAM and OAM are not conserved individually, and the evolution of SAM and OAM is relative to $v\hat{\boldsymbol{\tau}} \times \hat{\mathbf{p}}$, which is similar with the *curl* of energy flow density. However, the total angular momentum operator $\hat{\mathbf{J}} = \hat{\mathbf{L}} + \hat{\boldsymbol{\Sigma}}$ is conservative owing to

$$\dot{\hat{\mathbf{J}}} = \frac{i}{\hbar}\left[\hat{\mathbf{H}}, \hat{\mathbf{J}}\right] = \frac{i}{\hbar}\left[\hat{\mathbf{H}}, \hat{\mathbf{L}}\right] + \frac{i}{\hbar}\left[\hat{\mathbf{H}}, \hat{\boldsymbol{\Sigma}}\right] = 0 . \tag{S54}$$

The conservative properties of total angular momentum are critical in the analysis of the Chern number and spin-orbit interaction for structured electromagnetic waves.

**ii. Photonic spin Chern number for the structured electromagnetic waves**

The photonic spin Chern number for a plane wave is introduced in Ref. [9]. For a structured light field, the electric/magnetic field can be expressed in a plane-wave basis [s9]

$$\begin{aligned} \mathbf{E}(\mathbf{r}) &= \frac{1}{2\pi}\int_{|\mathbf{k}|=k} \tilde{\mathbf{E}}(\mathbf{k}) e^{i\mathbf{k}\cdot\mathbf{r}} d^2\mathbf{k} \\ \mathbf{H}(\mathbf{r}) &= \frac{1}{2\pi}\int_{|\mathbf{k}|=k} \tilde{\mathbf{H}}(\mathbf{k}) e^{i\mathbf{k}\cdot\mathbf{r}} d^2\mathbf{k} \end{aligned} , \tag{S55}$$

where k = ω/c = |**k**| is the wave number. Transversality constraints for plane waves given by the Maxwell's equations ∇·**E** = ∇·**H** = 0, which result in the requirements $\mathbf{k}\cdot\tilde{\mathbf{E}}(\mathbf{k}) = \mathbf{k}\cdot\tilde{\mathbf{H}}(\mathbf{k}) = 0$ for each single plane wave in the basis, relates the field vector to the wave vector and, therefore, reduces the full 3D vector space of the electromagnetic field components to the 2D subspace of the components tangential to a sphere in the **k** space. Owing to the conservation of total OAM Eq. (S54), this subspace is invariant for the total angular operator $\hat{\mathbf{J}}$, and one can divide it into two parts consistent with the transversality condition [s10]:

$$\hat{\mathbf{J}} = \hat{\mathbf{L}}' + \hat{\boldsymbol{\Sigma}}' ,\ \hat{\mathbf{L}}' = \hat{\mathbf{L}} - \boldsymbol{\kappa} \times \left(\boldsymbol{\kappa} \times \hat{\boldsymbol{\Sigma}}\right) \text{ and } \hat{\boldsymbol{\Sigma}}' = \boldsymbol{\kappa}\left(\boldsymbol{\kappa} \cdot \hat{\boldsymbol{\Sigma}}\right).$$

where **κ** = **k**/*k* and the modified OAM and SAM operators $\hat{\mathbf{L}}'$ and $\hat{\boldsymbol{\Sigma}}'$ can be regarded as projections of the operators $\hat{\mathbf{L}}$ and $\hat{\boldsymbol{\Sigma}}$ onto the transversality subspace.

Following Ref. [29], we choose an auxiliary vector $\mathbf{e}_0$ and define two unit vectors as

$$\mathbf{e}_2 = \frac{\mathbf{e}_0 \times \boldsymbol{\kappa}}{\left|\mathbf{e}_0 \times \mathbf{e}_\mathbf{k}\right|} \quad \mathbf{e}_1 = \mathbf{e}_2 \times \boldsymbol{\kappa} . \tag{S56}$$

The vectors ($\mathbf{e}_1$, $\mathbf{e}_2$, **κ**) form a Cartesian frame in which vectors $\tilde{\mathbf{E}}(\mathbf{k})$ and $\tilde{\mathbf{H}}(\mathbf{k})$ lie in the transverse plane ($\mathbf{e}_1$, $\mathbf{e}_2$). Next, we introduce the circular polarization basis:

$$\mathbf{e}_+(\mathbf{k}) = \frac{1}{\sqrt{2}}\left(\mathbf{e}_1 + i\mathbf{e}_2\right) \quad \mathbf{e}_-(\mathbf{k}) = \frac{1}{\sqrt{2}}\left(\mathbf{e}_1 - i\mathbf{e}_2\right), \tag{S57}$$

in which the plane-wave components of the field can be represented as

$$\tilde{\mathbf{E}}(\mathbf{k}) = \mathbf{C}_+(\mathbf{k}) + \mathbf{C}_-(\mathbf{k}) \tag{S58}$$

with $\mathbf{C}_\sigma(\mathbf{k}) = C_\sigma(\mathbf{k})\mathbf{e}_\sigma(\mathbf{k})$, where $\sigma$=±1 and $C_\sigma(\mathbf{k})$ are the scalar amplitudes of the circularly polarized components. For the structured waves, the OAM of the field is then

$$\mathbf{J} = \int \mathbf{r} \times (\mathbf{p}_s + \mathbf{p}_o) d^2\mathbf{r} = \mathbf{L} + \hat{\mathbf{\Sigma}} = \mathbf{L}' + \hat{\mathbf{\Sigma}}' , \tag{S59}$$

where the modulated SAM and OAM components are

$$\begin{aligned} \hat{\mathbf{\Sigma}}' &= \frac{1}{2\omega c}\sum_\sigma \int_{|\mathbf{k}|=k} \sigma\boldsymbol{\kappa}\left|\mathbf{C}_\sigma(\mathbf{k})\right|^2 d^2\mathbf{k} = \frac{1}{2\omega c}\int_{|\mathbf{k}|=k}\left[\left|\mathbf{C}_+(\mathbf{k})\right|^2 - \left|\mathbf{C}_-(\mathbf{k})\right|^2\right]\boldsymbol{\kappa} d^2\mathbf{k} \\ \mathbf{L}' &= \frac{1}{2\omega c}\sum_\sigma \int_{|\mathbf{k}|=k} \mathbf{C}^*_\sigma(\mathbf{k}) \cdot \left(-i\mathbf{k} \times \frac{\partial}{\partial \mathbf{k}} - \hat{\mathbf{A}}_\mathrm{B} \times \mathbf{k}\right)\mathbf{C}_\sigma(\mathbf{k}) d^2\mathbf{k} \end{aligned} . \tag{S60}$$

It is worth noting that the transformation to the helicity basis is associated with the transition to the local coordinate frame with the $z$ axis attached to the **k**-vector, which induces pure gauge Coriolis-type potential [s11]

$$\hat{\mathbf{A}}_\mathrm{B} = -i\hat{U}^\dagger \partial_\mathbf{k} \hat{U} . \tag{S61}$$

This is the Berry gauge field (connection), which corresponds to the monopole curvature $\hat{\mathbf{F}}_\mathrm{B} = \partial_\mathbf{k} \times \hat{\mathbf{A}}_\mathrm{B} = \hat{\sigma}\mathbf{k}/k^3$ with $\hat{\sigma} = \mathrm{diag}(1,-1,0)$ [s12]. By using the Dirac representation and electric–magnetic duality, it can be rewritten as

$$\begin{aligned} \mathbf{\Sigma}' &= \left\langle \Psi(\mathbf{k}) \middle| \hat{\sigma}\boldsymbol{\kappa} \middle| \Psi(\mathbf{k}) \right\rangle \\ \mathbf{L}' &= \left\langle \Psi(\mathbf{k}) \middle| \hat{\mathbf{L}} \middle| \Psi(\mathbf{k}) \right\rangle - \left\langle \Psi(\mathbf{k}) \middle| \hat{\sigma}\mathbf{A}_\mathrm{B} \times \mathbf{k} \middle| \Psi(\mathbf{k}) \right\rangle \end{aligned} . \tag{S62}$$

From this analysis, the topological Chern numbers for the two helical states can be defined as

$$C^\sigma = \frac{1}{2\pi}\int \left\langle \Psi(\mathbf{k}) \middle| \hat{\mathbf{F}}_\mathrm{B} \middle| \Psi(\mathbf{k}) \right\rangle d^2\mathbf{k} = \left\langle \Psi(\mathbf{k}) \middle| 2\sigma \middle| \Psi(\mathbf{k}) \right\rangle = 2\sigma , \tag{S63}$$

where the normalization condition, which has the meaning of the number of photons in the wave packet, has a form of $N = \left\langle \Psi(\mathbf{k}) \mid \Psi(\mathbf{k}) \right\rangle = 1$ [s10]. Therefore, we can obtain the total Chern number to be

$$C_t = \sum_{\sigma=\pm1} C^\sigma = 0 . \tag{S64}$$

The vanished total Chern number reflects the time-reversal symmetry of non-magnetic Maxwell surface modes.

On the other hand, the spin Chern number is

$$C_{spin} = \sum_{\sigma=\pm1} \sigma C^\sigma = 4 . \tag{S65}$$

This nonzero spin Chern number implies that the nontrivial helical states of electromagnetic waves indeed exist and are strictly locked to the energy propagation direction. Despite the existence of such nontrivial helical states at the interface governed by the spin-momentum locking, the topological $\mathbb{Z}_2$ invariant of these states vanishes

$$\upsilon = \frac{C_{spin}}{2} \bmod 2 = 0 \quad \text{(S66)}$$

owing to the time-symmetry of the Maxwell's equations. Thus, the spin-momentum locking of optical transverse spin discussed here is different from the "pseudo-spin" [6-8] in artificial photonic structures which is engineered to break the time-reversal symmetry, therefore, possessing protection against back-scattering. Although the transformation of the two helical states of evanescent waves are not topologically protected against scattering, the SML and the induced unidirectional excitation and transportation of photons are the intrinsic feature of the Maxwell's theory and are topological nontrivial (possess $\mathbb{Z}_4$ topological invariant).

**iii. Berry phase and spin texture for surface electromagnetic waves**

For the general case, the curl of energy flow given in Eq. (S12) can be abbreviated as

$$\nabla \times \mathbf{P} = \omega^2 \mathbf{S} - \frac{1}{2}\mathrm{Re}\left\{-\left(\nabla \otimes \mathbf{E}^*\right)_s \cdot \mathbf{H} + \left(\nabla \otimes \mathbf{H}^*\right)_s \cdot \mathbf{E}\right\}.$$

The second part of this equation has a similar structure as the quantum 2-form [33] that generates the Berry phase associated with a circuit, which indicates a spin-orbit interaction in the optical system. In the case of guided modes, this part can be rewritten as

$$\mathrm{Re}\left\{-\left(\nabla \otimes \mathbf{E}^*\right)_s \cdot \mathbf{H} + \left(\nabla \otimes \mathbf{H}^*\right)_s \cdot \mathbf{E}\right\} = 2\omega^2 \mathbf{S}. \quad \text{(S67)}$$

Thus, the phase change can be obtained as

$$\gamma(C) = \iint_C \mathrm{Re}\left\{-\left(\nabla \otimes \mathbf{E}^*\right)_s \cdot \mathbf{H} + \left(\nabla \otimes \mathbf{H}^*\right)_s \cdot \mathbf{E}\right\} \cdot d\mathbf{a} = \iint_C 2\omega^2 \mathbf{S} \cdot d\mathbf{a}, \quad \text{(S68)}$$

where $C$ is a two-dimensional connected region of the interface. By applying the general spin-momentum locking equation and the Stokes' theorem to Eq. (S68), the phase change can be rewritten as

$$\gamma(C) = \iint_C 2\omega^2 \mathbf{S} \cdot d\mathbf{a} = \iint \nabla \times \mathbf{P} \cdot d\mathbf{a} = \oint_{\mathbf{a}} \mathbf{P} \cdot d\mathbf{r}. \quad \text{(S69)}$$

Therefore, the phase change is determined by the optical trajectory around a connected space, which is analogous to the concept of geometric phase in condensed matter physics. In addition, this phase change, determined by the optical trajectory, can also be seen in the optical spin-Hall effect [s13] and optical Magnus effect [s12], which result in the separation of the two helical states and the helicity-depended unidirectional propagation. Thus, the momentum-locked chiral spin texture is also related to the optical-trajectory-determined Berry phase and spin-orbit interaction.

If we consider a region where the energy flow density vanishes at the boundary, the integral also vanishes. Thus, the orientation of SAM should be reversed at the two sides of the extreme point of the energy flow density. This can explain the chiral property of spin texture for the structured guided waves, which is one of the key points of our observations (Fig. 2 of the main text). In addition, the topological number of the chiral

spin texture can be +1/−1, which denote the spin vector varies from 'up' state to 'down' state or 'down' state to 'up' state accordingly.

Finally, if we consider the integral of the SAM over a total two-dimensional plane, we obtain

$$\iint_{a\to\infty} \mathbf{S}\cdot d\mathbf{a} = \frac{1}{2\omega^2}\oint_{a\to\infty} \mathbf{P}\cdot d\mathbf{r} = 0 \tag{S70}$$

owing to the disappearance of energy flow at infinity for a finite size optical beam. Equation (S70) confirms the "local" property of the transverse SAM of surface structured waves [26], in contrast to the longitudinal SAM, for which the integral does not vanish.

## V. Validation of the spin-momentum relation for the four structured surface waves considered in the experiment

We will now verify the above spin-momentum relation for various TM-polarised surface waves considered in the main text. Note that the TE mode evanescent waves can be verified in a same way by exchanging the electric field components with magnetic field components.

### i. Surface plane wave

For a monochromatic TM mode with an evanescent field decaying in $z$-direction, the $E_z$ field component satisfies the Helmholtz equation:

$$\nabla^2 E_z + k^2 E_z = 0\ , \tag{S71}$$

where $k = \omega/c$ is the wave vector of the wave. Assuming that the surface wave propagates along $y$-axis, the electric and magnetic fields can be written as [12]

$$\begin{aligned} \mathbf{E} &= \left(\hat{\mathbf{z}} - i\frac{k_z}{\beta}\hat{\mathbf{y}}\right)\exp\left[i\beta y - k_z z\right] \\ \mathbf{H} &= \frac{\omega\varepsilon}{\beta}\hat{\mathbf{x}}\exp\left[i\beta y - k_z z\right] \end{aligned}\ , \tag{S72}$$

where $\hat{\mathbf{x}}, \hat{\mathbf{y}}, \hat{\mathbf{z}}$ are the unit direction vectors. Here, $\beta = k_y$ is the propagation constant. Thus, the energy flow density of the evanescent wave is:

$$\mathbf{P} = \frac{1}{2}\mathrm{Re}\left\{\mathbf{E}^* \times \mathbf{H}\right\} = \frac{\omega\varepsilon}{2\beta}\hat{\mathbf{y}}\exp\left[-2k_z z\right]\ , \tag{S73}$$

and the SAM can be calculated to be:

$$\mathbf{S} = \frac{1}{4\omega}\mathrm{Im}\left[\varepsilon\left(\mathbf{E}^* \times \mathbf{E}\right) + \mu\left(\mathbf{H}^* \times \mathbf{H}\right)\right] = \frac{\varepsilon k_z}{2\omega\beta}\hat{\mathbf{x}}\exp\left[-2k_z z\right] = -\frac{1}{2\omega^2}\frac{\partial P_y}{\partial z}\ . \tag{S74}$$

By examining Eq. (S73) and Eq. (S74), one can find that the spin-momentum relationship for the surface plane wave is satisfied.

### ii. Surface Cosine beam

The same as above, assuming the beam propagates along *y*-direction, the *z*-component of the electric field can be expresses as [34]:

$$E_z = \mathrm{A}\cos(k_x x)\exp\left[ik_y y - k_z z\right], \tag{S75}$$

where A is a complex constant.

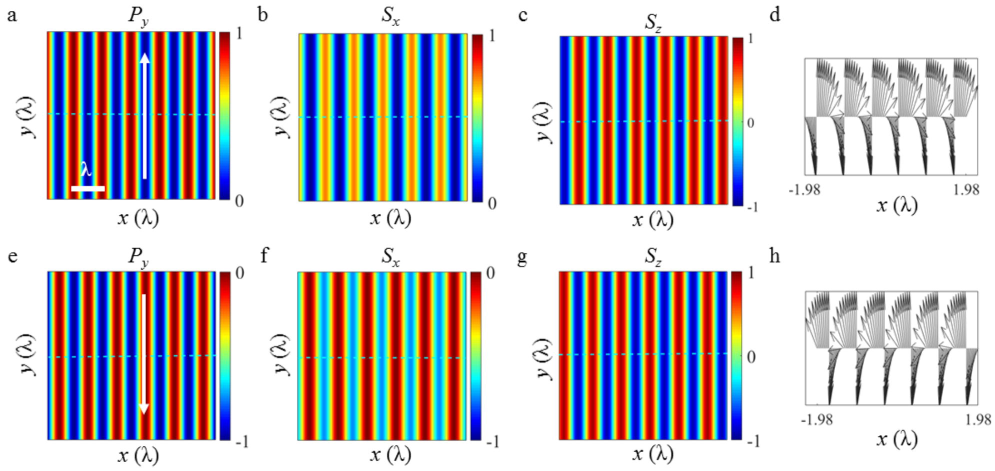


**Fig. S2. The Poynting vector and spin properties for the surface Cosine beams. a-c,** The energy flow density and SAM distributions for the surface Cosine beam propagating along the +y direction. **d**, The normalized spin vector pattern of the beam along the dashed lines in **a-c**. **e-g**, The energy flow density and SAM distributions for the surface Cosine beam propagating along the -y direction. **h**, the normalized spin vector pattern of the beam along the dashed lines in **e-g**. The arrows in (**a**) and (**e**) show the propagating direction of the beams. The scale bar and the distance unit are the wavelength of light in vacuum.

By employing Eq. (S13), the other field components can be calculated as

$$\begin{aligned} E_x &= \mathrm{A}\frac{k_x k_z}{\beta^2}\sin(k_x x)\exp\left[ik_y y - k_z z\right] \quad & H_x &= \mathrm{A}\frac{\omega\varepsilon k_y}{\beta^2}\cos(k_x x)\exp\left[ik_y y - k_z z\right] \\ E_y &= -\mathrm{A}\frac{ik_y k_z}{\beta^2}\cos(k_x x)\exp\left[ik_y y - k_z z\right] \quad & H_y &= -\mathrm{A}\frac{i\omega\varepsilon k_x}{\beta^2}\sin(k_x x)\exp\left[ik_y y - k_z z\right] \end{aligned}. \tag{S76}$$

The Poynting vector and the *curl* can be calculated as

$$\mathbf{P} = \frac{1}{2}\mathrm{Re}\left\{\mathbf{E}^* \times \mathbf{H}\right\} = |\mathrm{A}|^2 \frac{\omega\varepsilon k_y}{2\beta^2}\hat{\mathbf{y}}\cos^2(k_x x)\exp\left[-2k_z z\right] \tag{S77}$$

and

$$\nabla \times \mathbf{P} = |\mathrm{A}|^2 \frac{\omega\varepsilon k_y}{2\beta^2}\left\{\hat{\mathbf{x}}2k_z\cos^2(k_x x) - \hat{\mathbf{z}}k_x\sin(2k_x x)\right\}\exp\left[-2k_z z\right]. \tag{S78}$$

On the other hand, the SAM can be deduced to be:

$$\mathbf{S} = |\mathrm{A}|^2 \frac{\varepsilon k_y}{4\omega\beta^2}\left\{\hat{\mathbf{x}}2k_z\cos^2(k_x x) - \hat{\mathbf{z}}k_x\sin(2k_x x)\right\}\exp[-2k_z z] = \frac{1}{2\omega^2}\nabla\times\mathbf{P}\ , \tag{S79}$$

which satisfies Eq. (S23). The Poynting vector and SAM distributions for the Cosine beams with forward (+$y$ direction) and backward (–$y$ direction) propagation directions are summarized in Fig. S2, for the special case when $k_x = k_y = \beta\cdot\sin(\pi/4)$.

### iii. Surface Bessel beam

Surface Bessel beams are the solutions of the Maxwell's equations in the cylindrical coordinate ($r, \phi, z$). The general form of the $z$-component electric field can be expressed as [s14]:

$$E_z = \mathrm{A}\beta^2 J_l(\beta r)e^{il\phi}e^{-k_z z}, \tag{S80}$$

where $J_l$ stands for the Bessel function of the first kind with order $l$.

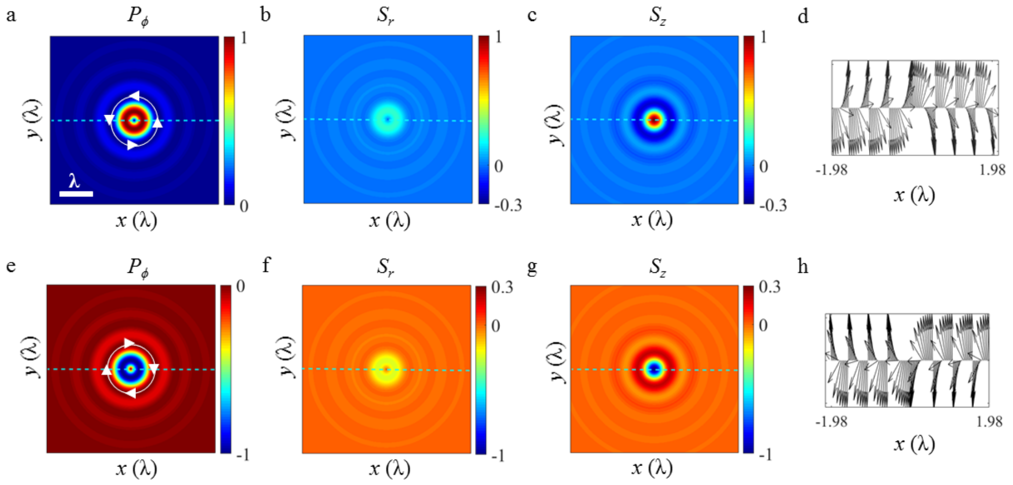


**Fig. S3. The Poynting vector and spin properties for the surface Bessel beams. a-c,** The energy flow density and SAM distributions for the surface Bessel beam with topological charge of +1, where the energy flow is counter-clockwise (as indicated by arrow in a). **d**, the normalized spin vector pattern of the beam along the dashed lines **a-c**. **e-g**, The energy flow density and SAM distributions for the surface Bessel beam with topological charge of -1, where the energy flow propagates clockwise (as indicated by arrow in e). **h**, the normalized spin vector pattern of the beam along the dashed lines in **e-g**. The scale bar and the distance unit are the wavelength of light in vacuum.

By employing Eq. (S13), the other field components can be calculated as:

$$
\begin{aligned}
E_r &= -\mathrm{A}k_z\beta J_l'(\beta r)e^{il\phi}e^{-k_z z} & H_r &= \mathrm{A}\frac{l\omega\varepsilon}{r}J_l(\beta r)e^{il\phi}e^{-k_z z} \\
E_\phi &= -\mathrm{A}\frac{ilk_z}{r}J_l(\beta r)e^{il\phi}e^{-k_z z} & H_\phi &= \mathrm{A}i\omega\varepsilon\beta J_l'(\beta r)e^{il\phi}e^{-k_z z} \\
E_z &= \mathrm{A}\beta^2 J_l(\beta r)e^{il\phi}e^{-k_z z} & H_z &= 0
\end{aligned}, \tag{S81}
$$

where $J_l'(\beta r)$ stands for the first order derivative of the Bessel function.

The Poynting vector and the *curl* can, therefore, be calculated as

$$
\mathbf{P} = \frac{1}{2}\mathrm{Re}\{\mathbf{E}^* \times \mathbf{H}\} = |\mathrm{A}|^2 \beta^2 \frac{l\omega\varepsilon}{2r} J_l^2(\beta r)\exp[-2k_z z]\hat{\mathbf{e}}_\phi \tag{S83}
$$

and

$$
\nabla \times \mathbf{P} = |\mathrm{A}|^2 \frac{l\omega\varepsilon\beta^2}{r}\{\hat{\mathbf{e}}_r k_z J_l^2(\beta r) + \hat{\mathbf{e}}_z \beta J_l(\beta r) J_l'(\beta r)\}\exp[-2k_z z]. \tag{S84}
$$

Here, $\hat{\mathbf{e}}_r, \hat{\mathbf{e}}_\phi, \hat{\mathbf{e}}_z$ are the unit direction vectors of the cylindrical coordinate system. On the other hand, the SAM can be represented as

$$
\mathbf{S} = |\mathrm{A}|^2 \frac{l\varepsilon\beta^2}{2\omega r}\{\hat{\mathbf{e}}_r k_z J_l^2(\beta r) + \hat{\mathbf{e}}_z \beta J_l(\beta r) J_l'(\beta r)\}\exp[-2k_z z] = \frac{1}{2\omega^2}\nabla \times \mathbf{P}, \tag{S85}
$$

which satisfies Eq. (S23). The Poynting vector and SAM distributions for the surface Bessel beams with topological charge of $l$= +/−1 are given in Fig. S3.

**iv. Surface Weber beam**

Surface Weber beams are the solutions of the Maxwell's equations in the cylinder parabolic coordinates with a trial solution of $E_z = F(\sigma)G(\tau)e^{-k_z \cdot z}$, where $F$ and $G$ are the separation functions of $\sigma$ and $\tau$. In the parabolic coordinates $(\sigma, \tau, z)$, where $\sigma \in (-\infty, \infty); \tau \in [0, \infty); z \in (0, \infty)$, the coordinates are related to those in the Cartesian coordinates by: $x+iy=(\sigma+i\tau)^2/2$ and $z = z$.

By substituting the trial solution into the Helmholtz equation, one can get a characteristic formula for the surface Weber beam as:

$$
\frac{1}{F(\sigma)}\left[\frac{\partial^2 F(\sigma)}{\partial\sigma^2} + \sigma^2\beta^2 F(\sigma)\right] + \frac{1}{G(\sigma)}\left[\frac{\partial^2 G(\tau)}{\partial\tau^2} + \tau^2\beta^2 G(\tau)\right] = 0. \tag{S86}
$$

Since the two terms are functions of independent variables $\sigma$ and $\tau$, they can be separated as:

$$
\begin{aligned}
&\frac{\partial^2 F(\sigma)}{\partial\sigma^2} + (\sigma^2\beta^2 + 2\beta a)F(\sigma) = 0 \\
&\frac{\partial^2 G(\tau)}{\partial\tau^2} + (\tau^2\beta^2 - 2\beta a)G(\tau) = 0
\end{aligned}, \tag{S87}
$$

where $2\beta a$ is the separation constant. Eq. (S87) demonstrates a two-dimensional propagation-invariant Weber beams in a parabolic cylindrical coordinate [s14].

After solving the equation and transferring back to the Cartesian coordinates and using the relationship: $x = \sigma\tau$, $y = (\sigma^2 - \tau^2)/2$, the general form of the propagating-wave solution is

$$\begin{aligned} E_z &= \frac{1}{\sqrt{2\pi}}\left[|\Gamma_1|^2 \mathrm{F}_e(\sigma)\mathrm{G}_e(\tau) + 2i|\Gamma_3|^2 \mathrm{F}_o(\sigma)\mathrm{G}_o(\tau)\right]e^{-k_z z} \\ &= \frac{1}{\sqrt{2\pi}} e^{-i\frac{\beta(\sigma^2+\tau^2)}{2}-k_z z}\left\{\begin{array}{l} |\Gamma_1|^2\, {}_2\mathrm{F}_1\left[\frac{ia}{2}+\frac{1}{4};\frac{1}{2};i\beta\sigma^2\right]{}_2\mathrm{F}_1\left[-\frac{ia}{2}+\frac{1}{4};\frac{1}{2};i\beta\tau^2\right] \\ +2i|\Gamma_3|^2\beta\sigma\tau\, {}_2\mathrm{F}_1\left[\frac{ia}{2}+\frac{3}{4};\frac{3}{2};i\beta\sigma^2\right]{}_2\mathrm{F}_1\left[-\frac{ia}{2}+\frac{3}{4};\frac{3}{2};i\beta\tau^2\right] \end{array}\right\}, \end{aligned} \quad \text{(S88)}$$

where $\Gamma_1 = \Gamma[ia/2 + 1/4]$, $\Gamma_3 = \Gamma[ia/2 + 3/4]$ and Γ[x] is the complex Gamma function. Here, $F_1$ is the confluent hypergeometric function of the first kind. The Poynting vector and SAM distributions for the evanescent Weber beams propagating along +/−*y* directions are illustrated in Fig. S4 for the beam parameter *a*=40. Note that we use the numerical calculation to solve the field components of a Weber beam with Eq. (S13).

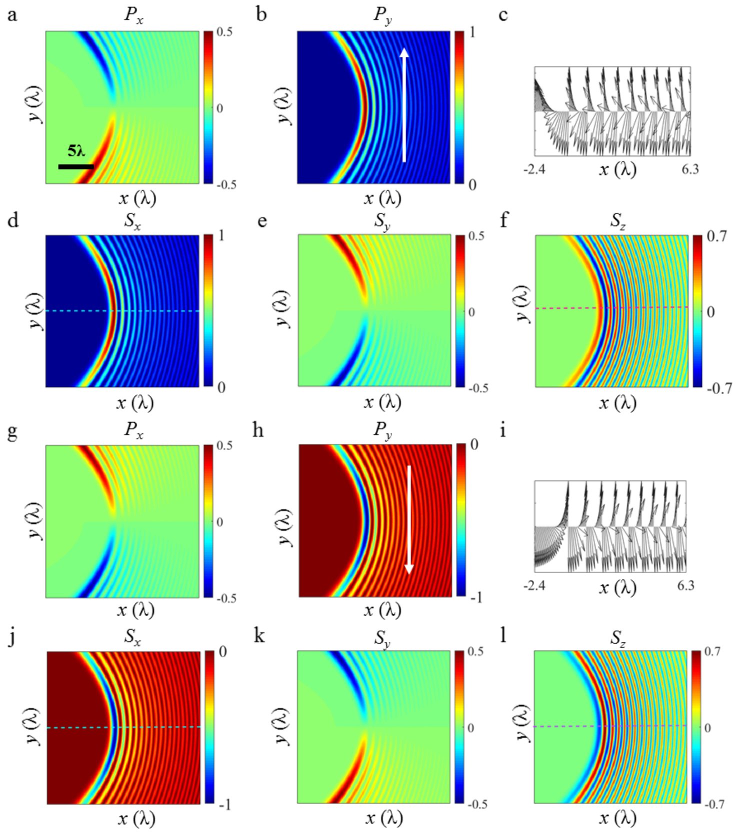


**Fig. S4. The Poynting vector and spin properties for the surface Weber beams. a-f,** The energy flow density (a,b) and SAM distributions (d-f) for the surface Weber beam propagating along the +y direction. **c**, The normalized spin vector pattern of the beam along the dashed lines in **f**. **g-l**, The energy flow density (g,h) and SAM distributions (j-l) for the surface Weber beam propagating along the -y direction. **i**, the normalized spin vector pattern of the beam along the dashed lines in **l**. The arrows in (**b**) and (**h**) show the

propagation direction of the Weber beams. The scale bar and the distance unit are the wavelength of light in vacuum.

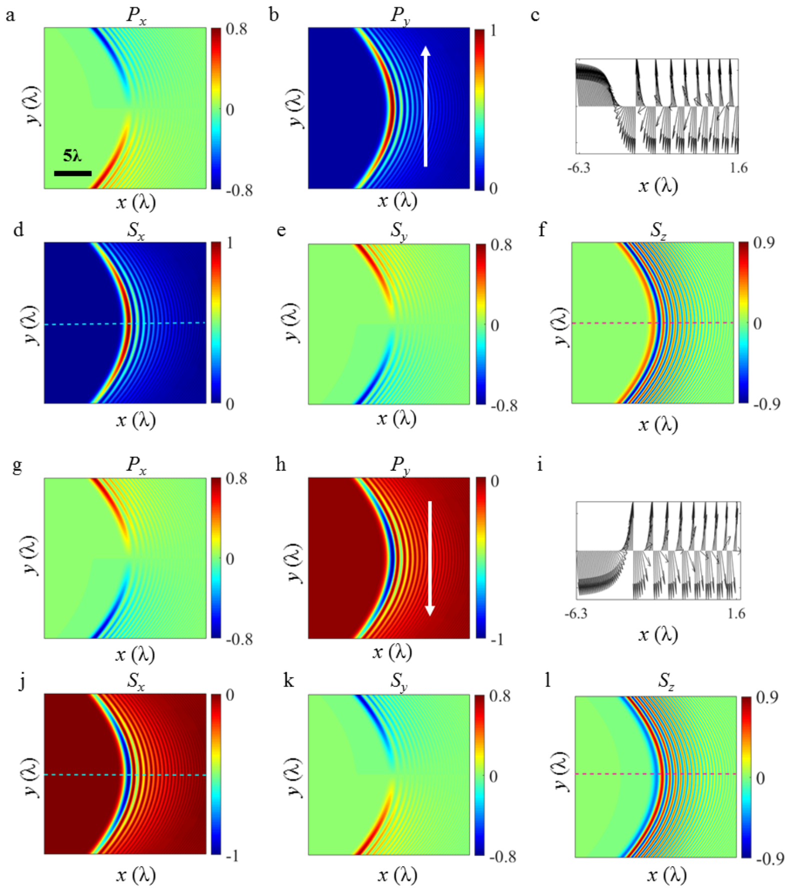


**Fig. S5. The Poynting vector and spin properties for the surface Airy beams. a-f,** The energy flow density (a,b) and SAM distributions (d-f) for the surface Airy beam propagating in the +y direction. **c**, the normalized spin vector pattern of the beam along the dashed lines in **f**. **g-l**, The energy flow density (g,h) and SAM distributions (j-l) for the surface Airy beam propagating in the −y direction. **i**, the normalized spin vector pattern of the beam along the dashed lines in **l**. The arrows in (**b**) and (**h**) show the propagating direction of the Airy beams. The scale bar and the distance unit are the wavelength of light in vacuum.

**v. Surface Airy wave**

By employing the trial solution of $E_z = \mathrm{A}(x,y)e^{ik_y y - k_z z}$, the Helmholtz equation can be simplified to $\frac{\partial^2 \tilde{A}}{\partial x^2} + 2i\beta\frac{\partial \tilde{A}}{\partial y} = 0$ , when the small quantity $\frac{\partial^2 \tilde{A}}{\partial y^2}$ representing the variation of the field along the

propagating direction can be ignored under the paraxial approximation $\sqrt{\frac{\ln 2}{(aw_m)^2}} \ll \beta$. The solution of the Airy function $\frac{\partial^2 \tilde{A}}{\partial x^2} + 2i\beta \frac{\partial \tilde{A}}{\partial y} = 0$ can be expressed as [s15]

$$\tilde{A}(x,y) = \mathrm{Ai}\left(x_m - \frac{y_m^2}{4} + iay_m\right) e^{i\left[\frac{1}{2}\left(x_m + a^2\right)y_m - \frac{1}{12}y_m^3\right]} e^{a\left(x_m - \frac{1}{2}y_m^2\right)}, \tag{S89}$$

where Ai indicates the Airy function of the first kind, $x_m = x/w_m$ and $y_m = y/\beta w_m^2$ are the modulated coordinates, $a$ is a parameter defining the exponential apodization of the Airy beam, and $2w_m$ is the width of the main lobe.

The Poynting vector and SAM distributions for the surface Airy beams propagating along the +/−y-direction are summarized in Fig. S5 for the beam parameters $a = 0.01$ and $w_m = 1.1\pi/\beta$.

The summary for the above four types of surface structured beams considered in experiments are shown in Table. S1. Note that we use the numerical simulations to solve the field components of the Weber beams with Eq. (S13), as the derivations of the confluent hypergeometric functions are unknown. Therefore, the analytical expressions of the energy flow density and the SAM for the Weber beams are not provided in Table. S1.

**Table. S1. Summary of $E_z$, energy flow density (EFD) and SAM for surface Cosine, Bessel, Weber and Airy beams.**

| Sum | Cosine beam | Bessel beam | Weber beam | Airy beam |
|---|---|---|---|---|
| **Helmholtz equation** | $\nabla^2 E_z + k^2 E_z = 0$ | | | |
| **Trial solution** | $A(x,y)e^{-k_z z}$ | $A(r,\phi)e^{-k_z z}$ | $F(\sigma)G(\tau)e^{-k_z \cdot z}$ | $A(x,y)e^{ik_y y - k_z z}$ |
| $\boldsymbol{E_z}$ | $cos(k_x x)e^{ik_y y - k_z z}$ | $k_r^2 J_l(k_r r)e^{il\phi - k_z z}$ | $\frac{1}{\sqrt{2}\pi}[\lvert\Gamma_1\rvert^2 F_e(\sigma)G_e(\tau) + 2i\lvert\Gamma_3\rvert^2 F_o(\sigma)G_o(\tau)]e^{-k_z \cdot z}$ | $\frac{1}{\varepsilon}\tilde{A}(x,y)e^{ik_y y - k_z z}$ |
| **EFDs** | $\hat{\mathbf{y}}C cos^2(k_x x)$ | $\hat{\boldsymbol{\phi}} C J_l^2(k_r r)$ | $\frac{1}{2}\{-Re(E_z^{d*}H_y^d)\hat{\mathbf{x}} + Re(E_z^{d*}H_x^d)\hat{\mathbf{y}}\}$ | $\left\{\begin{array}{c} \hat{\mathbf{x}} C \mathrm{Im}\left(\tilde{A}\frac{\partial \tilde{A}^*}{\partial x}\right) \\ +\hat{\mathbf{y}} C\left[\mathrm{Im}\left(\tilde{A}\frac{\partial \tilde{A}^*}{\partial y}\right) - ik_r \lvert\tilde{A}\rvert^2\right]\end{array}\right\}$ |
| **SAMs** | $\frac{C}{2\omega^2}\{-\hat{\mathbf{z}}k_x sin(2k_x x) + \hat{\mathbf{x}}2k_z cos^2(k_x x)\}$ | $\frac{C}{\omega^2}\{\hat{\mathbf{r}}k_z J_l^2(k_r r) + \hat{\mathbf{z}}k_r J_l(k_r r)J'_l(k_r r)\}$ | $\frac{i}{2\omega}\left\{\begin{array}{c} Im(\varepsilon E_z^{d*}E_y^d)\hat{\mathbf{x}} \\ Im(\varepsilon E_z^d E_x^{d*})\hat{\mathbf{y}} \\ Im[\varepsilon E_y^{d*}E_x^d + \mu H_x^d H_y^{d*}]\hat{\mathbf{z}}\end{array}\right\}$ | $\frac{C}{\omega^2}\left\{\begin{array}{c} \hat{\mathbf{x}}k_z\left[\mathrm{Im}\left(\tilde{A}\frac{\partial \tilde{A}^*}{\partial y}\right) - ik_r\lvert\tilde{A}\rvert^2\right] \\ -\hat{\mathbf{y}}k_z \mathrm{Im}\left(\tilde{A}^*\frac{\partial \tilde{A}}{\partial x}\right) \\ +\hat{\mathbf{z}}\left[\mathrm{Im}\left(\frac{\partial \tilde{A}}{\partial x}\frac{\partial \tilde{A}^*}{\partial y}\right) + ik_r \mathrm{Re}\left(\tilde{A}\frac{\partial \tilde{A}^*}{\partial x}\right)\right]\end{array}\right\}$ |
| **Parameter** | $C = \frac{\omega\varepsilon k_y}{2k_r^2}e^{-2k_z z}$ | $C = \frac{\omega\varepsilon l}{2r}k_r^2 e^{-2k_z z}$ | $y + ix = (\sigma + i\tau)^2/2$; $\sigma \in (-\infty, \infty); \tau \in [0, \infty)$ | $C = \frac{i\omega}{2\varepsilon k_r^2}e^{-2k_z z}$ |

## VI. Discussion on spin/momentum locking feature for surface electromagnetic waves

The demonstrated spin-momentum *curl* relation exhibits the intrinsic locking property between the SAM and optical energy flow density, and extends the spin-momentum locking to an arbitrary structured guided wave. This is one of the interesting physical effects as demonstrated in the main text. Moreover, starting from this relationship and noting that the Poynting vector of the field can be divided into the spin (**P**$_s$) and orbital part (**P**$_o$): **P**=**P**$_s$+**P**$_o$, where **P**$_s$=$v^2\nabla\times$S/2, we can obtain a set of spin/momentum equations that are analogous to the Maxwell equations (Table. 1 in the main text). As mentioned in the main text, one can obtain the spin and orbital properties of the guided electromagnetic waves directly from the spin/momentum equations without any knowledge about the electric and magnetic fields.

In a traditional manner, the spin and orbital angular momentum properties of an electromagnetic field is obtained by firstly calculating the electric and magnetic fields. For a time-harmonic monochromatic electromagnetic wave in a source free, homogeneous and linear isotropic medium, the Hertz's wave equation independent of the coordinate system is [s6]

$$\nabla^2\mathbf{\Pi}+k^2\mathbf{\Pi}=0\ . \quad \text{(S90)}$$

Eq. (S90) has two types of independent solutions: $\mathbf{\Pi}_e$ and $\mathbf{\Pi}_m$, where the subscript "*e*" and "*m*' denote for the TM and TE mode electromagnetic waves, respectively. These result in independent sets of TM waves

$$\begin{aligned}\mathbf{E}&=k^2\mathbf{\Pi}_e+\nabla\left(\nabla\cdot\mathbf{\Pi}_e\right)\\ \mathbf{H}&=-i\omega\varepsilon\nabla\times\mathbf{\Pi}_e\end{aligned}, \quad \text{(S91)}$$

and TE waves

$$\begin{aligned}\mathbf{E}&=i\omega\varepsilon\nabla\times\mathbf{\Pi}_m\\ \mathbf{H}&=k^2\mathbf{\Pi}_m+\nabla\left(\nabla\cdot\mathbf{\Pi}_m\right)\end{aligned}. \quad \text{(S92)}$$

Assuming the optical axis along the z-direction so that $\mathbf{\Pi}$ can be expressed as $\mathbf{\Pi}=\Psi\hat{\mathbf{z}}$, where $\Psi$ is the model of vector Hertz potential. Obviously, the Hertz potential satisfies the Helmholtz equation:

$$\nabla^2\Psi+k^2\Psi=0\ . \quad \text{(S93)}$$

For the TM waves, the electric and magnetic fields can be calculated as:

$$\begin{aligned}E_x&=-k_z\frac{\partial\Psi}{\partial x} & H_x&=-i\omega\varepsilon\frac{\partial\Psi}{\partial y}\\ E_y&=-k_z\frac{\partial\Psi}{\partial y} & H_y&=i\omega\varepsilon\frac{\partial\Psi}{\partial x}\\ E_z&=\left(k^2+k_z^2\right)\Psi & H_z&=0\end{aligned}\ . \quad \text{(S94)}$$

and for the TE waves, they are:

$$\begin{aligned}E_x&=i\omega\mu\frac{\partial\Psi}{\partial y} & H_x&=-k_z\frac{\partial\Psi}{\partial x}\\ E_y&=-i\omega\mu\frac{\partial\Psi}{\partial x} & H_y&=-k_z\frac{\partial\Psi}{\partial y}\\ E_z&=0 & H_z&=\left(k^2+k_z^2\right)\Psi\end{aligned}\ , \quad \text{(S95)}$$

After obtaining the electromagnetic field, one can calculate the Poynting vector, spin and orbital angular momentum by the classic definition as in Eq. (S1) and Eq. (S7).

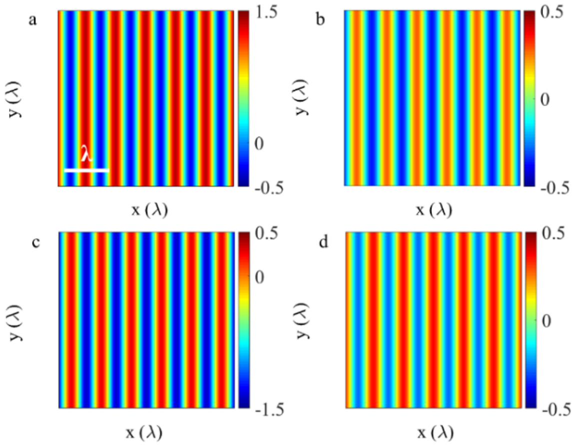


**Fig. S6. The orbital and spin flow density for the surface Cosine mode.** **a** and **b** show the $\mathbf{P}_o$ and $\mathbf{P}_s$ for the surface Cosine mode propagating in +y direction with $k_x = \beta \cdot \sin(\pi/4)$. **c** and **d** give the $\mathbf{P}_o$ and $\mathbf{P}_s$ for the surface Cosine mode propagating in −y direction with $k_x = \beta \cdot \sin(\pi/4)$. All quantities are normalized by the maximum of energy flow density.

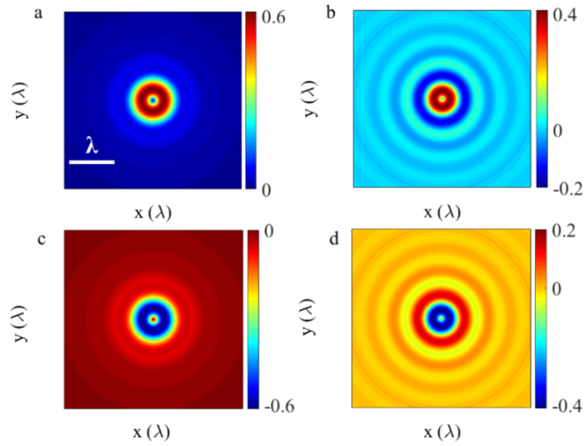


**Fig. S7. The orbital and spin flow density for the surface Bessel mode.** **a** and **b** show the $\mathbf{P}_o$ and $\mathbf{P}_s$ for the surface Bessel mode propagating in +φ direction. **c** and **d** indicate the $\mathbf{P}_o$ and $\mathbf{P}_s$ for the surface Bessel mode propagating in −φ direction. All quantities are normalized by the maximum of energy flow density.

Noting that the Poynting vector can be calculated from the Hertz potential directly by $\mathbf{P} \propto (\Psi^*\nabla\Psi - \Psi\nabla\Psi^*)$, we can obtain the spin and orbital properties of the guided electromagnetic waves directly from the Maxwell's equations without any knowledge about the electric and magnetic fields. It is worth to mention that the classification of the electromagnetic field to the TM and TE modes in this case, becomes unnecessary because they have the same spin and orbital properties. In this way, we can obtain the orbital flow density $\mathbf{P}_o = \mathbf{P} + \mathbf{\Delta P}/4k^2$ and spin flow density $\mathbf{P}_s = -\mathbf{\Delta P}/4k^2$, where $\mathbf{\Delta}$ is the Laplace operator. The spatial distributions of $\mathbf{P}_o$ and $\mathbf{P}_s$ for the four surface structured waves are shown in Figs. S6-S9, respectively.

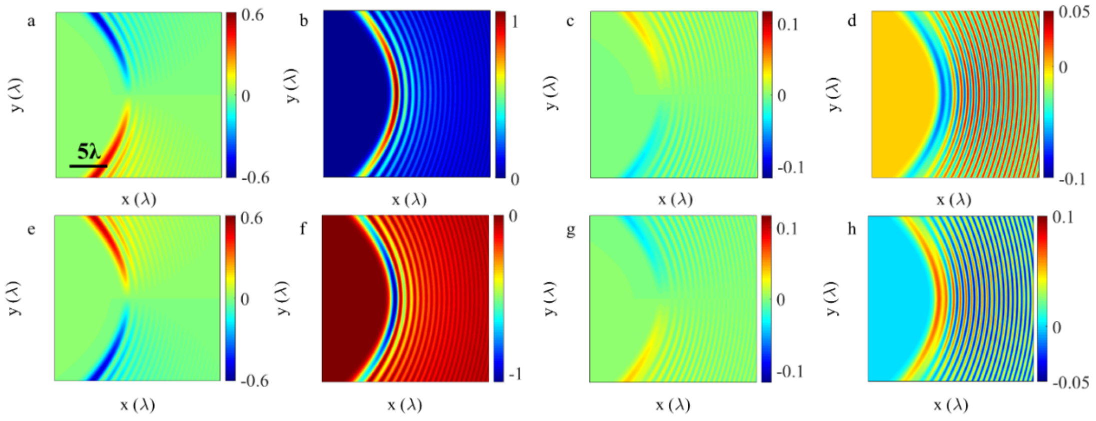


**Fig. S8. The orbital and spin flow density for the surface Weber mode. a**, **b** show the x and y-components of $\mathbf{P}_o$ and **c**, **d** exhibit the x and y-components of $\mathbf{P}_s$ for the surface Weber mode propagating in +y direction with $a$ = 40. **e**, **f** show the x and y-components of $\mathbf{P}_o$ and **g**, **h** exhibit the x and y-components of $\mathbf{P}_s$ for the surface Weber mode propagating in −y direction with $a$ = 40. All quantities are normalized by the maximum of energy flow density.

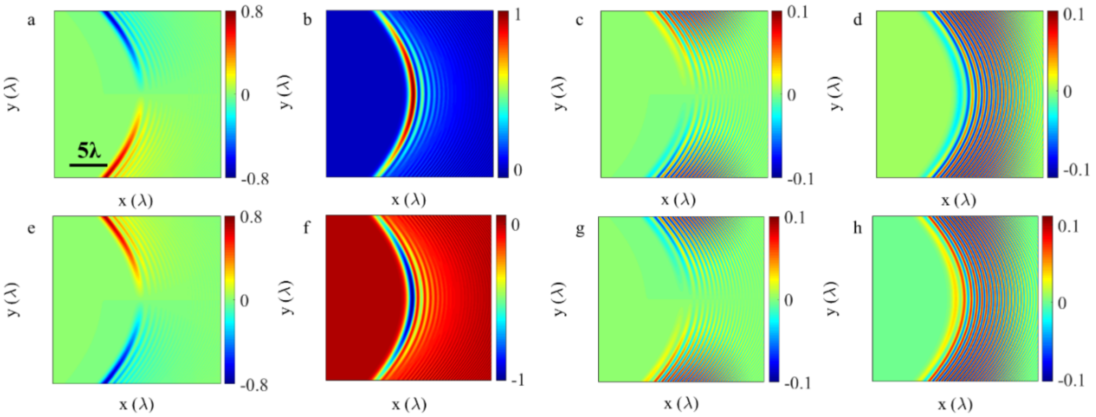


**Fig. S9. The orbital and spin flow density for the surface Airy mode. a**, **b** show the x and y-components of $\mathbf{P}_o$ and **c**, **d** exhibit the x and y-components of $\mathbf{P}_s$ for the surface Airy mode propagating in +y direction with $a$ = 0.01. **e**, **f** show the x and y-components of $\mathbf{P}_o$ and **g**, **h** exhibit the x and y-components of $\mathbf{P}_s$ for the surface Airy mode propagating in −y direction with $a$ = 0.01. All quantities are normalized by the maximum of energy flow density.

## VII. Experimental setup and methods

The experimental setup is shown in **Fig. S10**. The experiment was performed on the example of SPPs, which are TM mode evanescent waves supported at a metal- dielectric interface. A He-Ne laser beam with a

wavelength of 632.8nm was used as a light source. After a telescope system to expand the beam, a combination of linear polarizer (LP), half-wave plates (HWP), quarter-wave plates (QWPs) and vortex wave plates (VWPs) was employed to modulate the polarization of the laser beam. A spatial light modulator (SLM) was then utilized to modulate the phase of the beam.

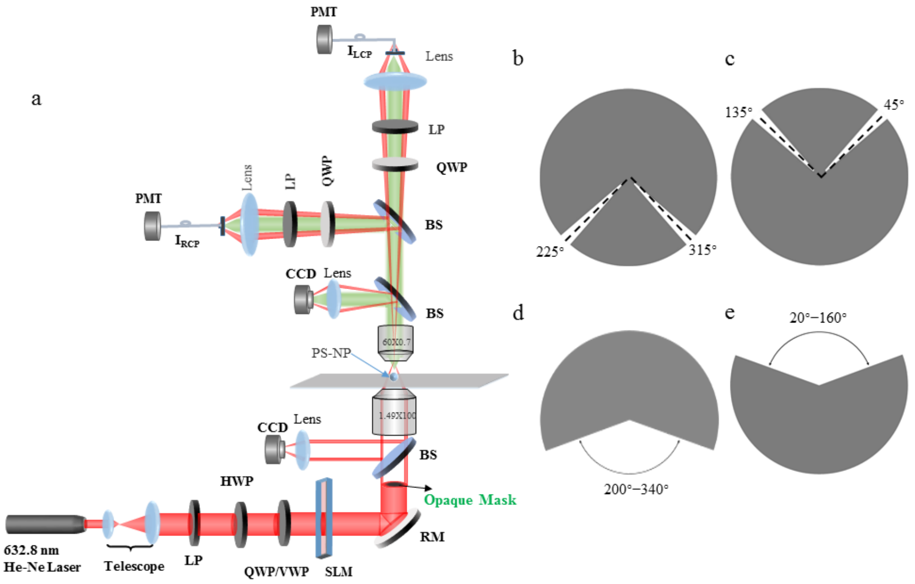


**Fig. S10. The experimental setup for excitation and mapping of the structured SPP waves**. **a**, Schematic diagram. **b-c**, The designed opaque masks employed in the experiment for generation of a SPP Cosine beams with opposite propagation directions. Incident light was blocked except for the two open segments with the angle spread of 8°. **d**, The designed opaque mask for generation of SPP Weber and Airy beams. Note that a spatial light modulator (SLM) was also employed in order to generate the desired Weber beam and Airy beam. **e**, the mask complementary of (**d**) to generate the corresponding beams with opposite propagation directions to those generated with (d). LP: linear polarizer; HWP: half-wave plate; QWP: quarter-wave plate; VWP: vortex wave plate; RM: reflector mirror; PMT: photo-multiplier tube; BS: non-polarizing beam-splitter.

The structured beam was then tightly focused by an oil-immersion objective (Olympus, NA=1.49, 100×) onto the sample consisting of a thin silver film (45-nm thickness) deposited on a cover slip, to form the desired SPP beams at the air/silver interface. A polystyrene nanosphere of 160 nm radius was immobilized on the silver film surface, as a near-field probe to scatter the SPPs to the far field. The preparation of the sample can be found elsewhere [s16]. The sample was fixed on a Piezo scanning stage (Physik Instrumente, P-545) providing resolution down to 1 nm. A low NA objective (Olympus, NA=0.7, 60×) was employed to collect the scattering radiation from the nanosphere. A combination of quarter wave plate (QWP) and linear polarizer was used to extract the right-handed (RCP) and left-handed (LCP) circular polarization components of the collected signals. Finally, the intensities of RCP and LCP components are measured by a photo-multiplier

tube (PMT, Hamamatsu R12829). As the piezo scanning stage raster scanned the near-field region, the distributions of RCP and LCP components can be mapped and used to reconstruct the longitudinal SAM component: $S_z = (I_{RCP} - I_{LCP})\varepsilon\beta^2/4\omega k_z^2$.

### i. Surface Cosine wave

To generate the SPP Cosine beams in the experiment, a pair of opaque masks were designed and put right below the objective lens in order to filter the wave vectors in the incident plane of the objective. Incident light was, thus, blocked except for the two open angles with angle spread of 8$^o$ (**Fig. S10**(b-c)).

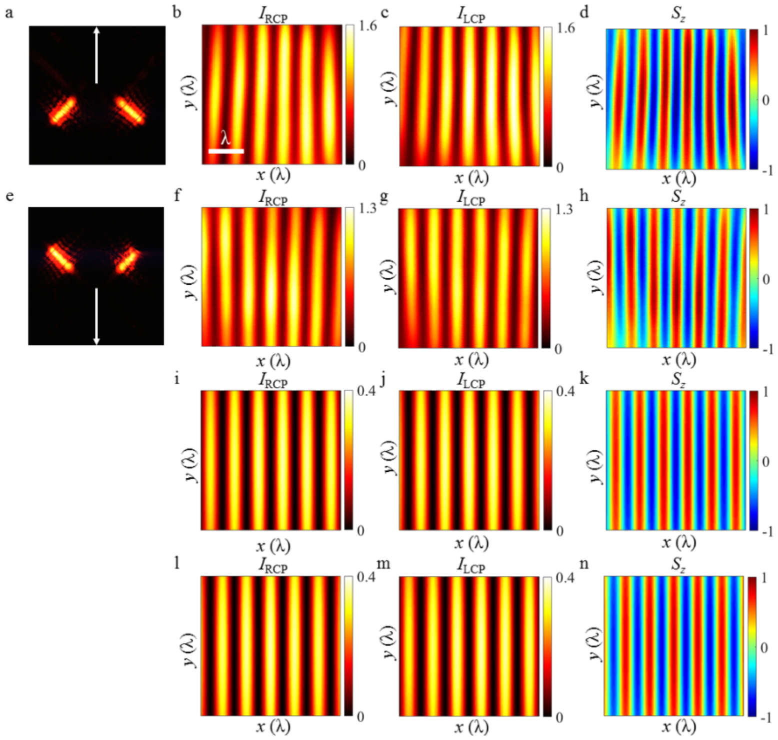


**Fig. S11. Experimental results for the SPP Cosine beams. a**, The image of the reflected beam captured at the back focal plane of the excitation objective lens when the opaque mask shown in **Fig. S10**(b) was used. In this case, an SPP Cosine beam propagating along the +y direction was generated. **b-d**, The measured intensity distributions of the RCP and LCP components and the retrieved distribution of the longitudinal SAM ($S_z$) component of the SPP Cosine beam. **e-h**, The same as b-d for the opaque mask shown in **Fig. S10**(c) which generates an SPP Cosine beam propagating along the –y direction. The scanning step size in the experiment was 25 nm. The reversal of the optical spin for the two Cosine beams with opposite propagation directions is clearly observed. The arrows in (**a**) and (**e**) show the propagating direction of the generated Cosine beams. **i-k** and **l-n**, The corresponding theoretical calculation results obtained with the vectorial diffraction theory for b-d and e-h, respectively. The distance units are the wavelength of light in vacuum.

The experimental results are shown in **Fig. S11**(a-h) together with the simulation results in Fig. **S11**(i-n) obtained with the vectorial diffraction theory [s17]. As can be seen, the two opaque masks generate the SPP Cosine beams with opposite propagation directions. The theoretical and experimental results match well and the spin-momentum locking property of the SPP Cosine beams is clearly demonstrated.

**ii. Surface Bessel wave**

The method to generate the SPP Bessel beams can be found elsewhere [35]. Here, we use the left-handed and right-handed CP lights to generate the SPP Bessel beams with topological charge of +1 and –1, respectively. The experimental results are shown in **Fig. S12**, along with the theoretical results for comparison. The spin-momentum locking property for the SPP Bessel beams was clearly demonstrated.

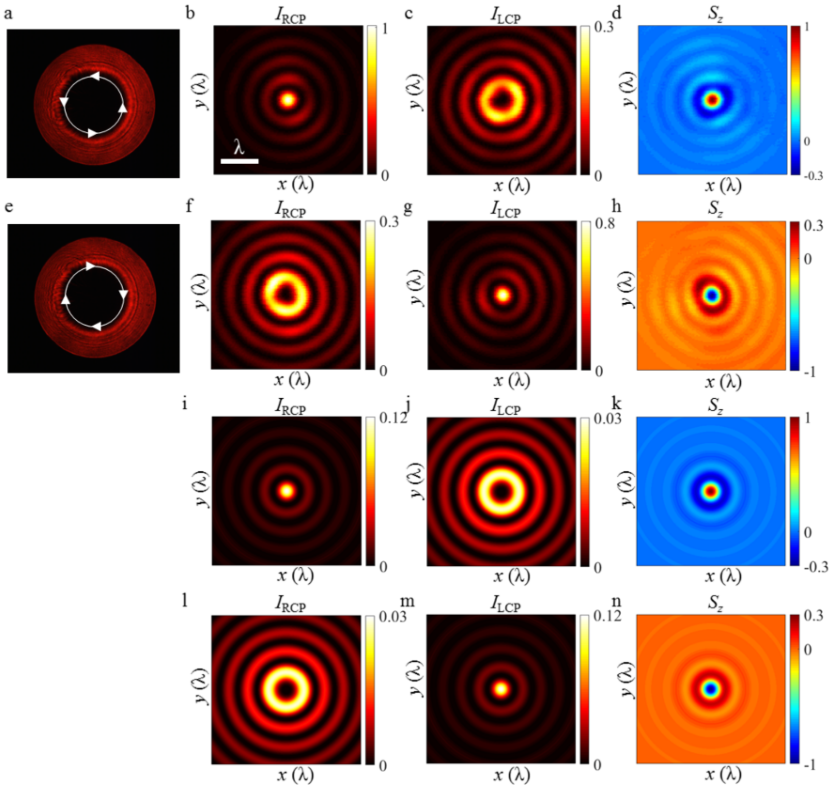


**Fig. S12. Experimental results for the SPP Bessel beams. a**, The back focal plane image of the reflected beam when LCP light was used to the excite the SPP with topological charge of +1 and the energy propagating counter-clockwise. **b-d**, The mapped $I_{RCP}$ and $I_{LCP}$ distributions and the retrieved distribution of the $S_z$ component of the SPP Bessel beam. **e-h**, The same as b-d for RCP light used to excite the SPP Cosine beam propagating clockwise. The scanning step size in the experiment was 20 nm. It clearly demonstrates the reversal of the optical spin for the two Bessel beams with opposite propagation directions. The arrows in (**a**) and (**e**) indicate the propagating directions of the generated Bessel beams. **i-k** and **l-n**, The corresponding theoretical calculation results obtained with the vectorial diffraction theory for b-d and e-h, respectively. The distance units are the wavelength of light in vacuum.

### iii. Surface Weber and Airy beams

The SPP Weber and Airy beams were generated by the vectorial Fourier integral method [s15]. In the experiment, we utilize the opaque masks as shown in **Figs. S10**(d-e) to adjust the spatial frequencies of incident light and employ the SLM to code the phases into the incident beam. The phase for the generation of SPP Weber wave can be expressed as

$$\psi_{Weber} = ia\,k_y/k_r + ia\ln\left(\tan\left(\cos^{-1}\frac{k_y/k_r}{2}\right)\right) \tag{S96}$$

with a parameter $a$=10 was used for the experiment. The phase diagram shown in **Fig. S13**(a), together with the opaque mask shown in **Fig. S10**(d), was employed to generate the SPP Weber beam propagating in +y direction. Similarly, the phase diagram shown in **Fig. S13**(b) and the mask shown in **Fig. S10**(e) were employed to generate the Weber beam propagating in –y direction. The experimental results along with the theoretical simulations are shown in **Fig. S14**.

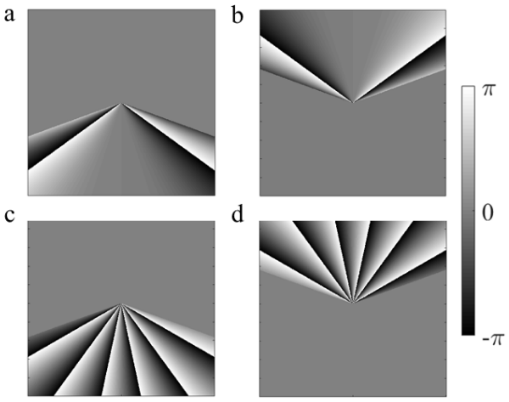


**Fig. S13. Phase diagrams produced with the SLM for generation of the SPP Weber and Airy beams**. **a-b**, the phases used for generation of the SPP Weber beams with opposite propagating directions: **a** for the +y direction and **b** for the –y direction. **c-d**, the phases for generation of the SPP Airy beams with opposite propagating directions: **c** for the +y direction and **d** for the –y direction.

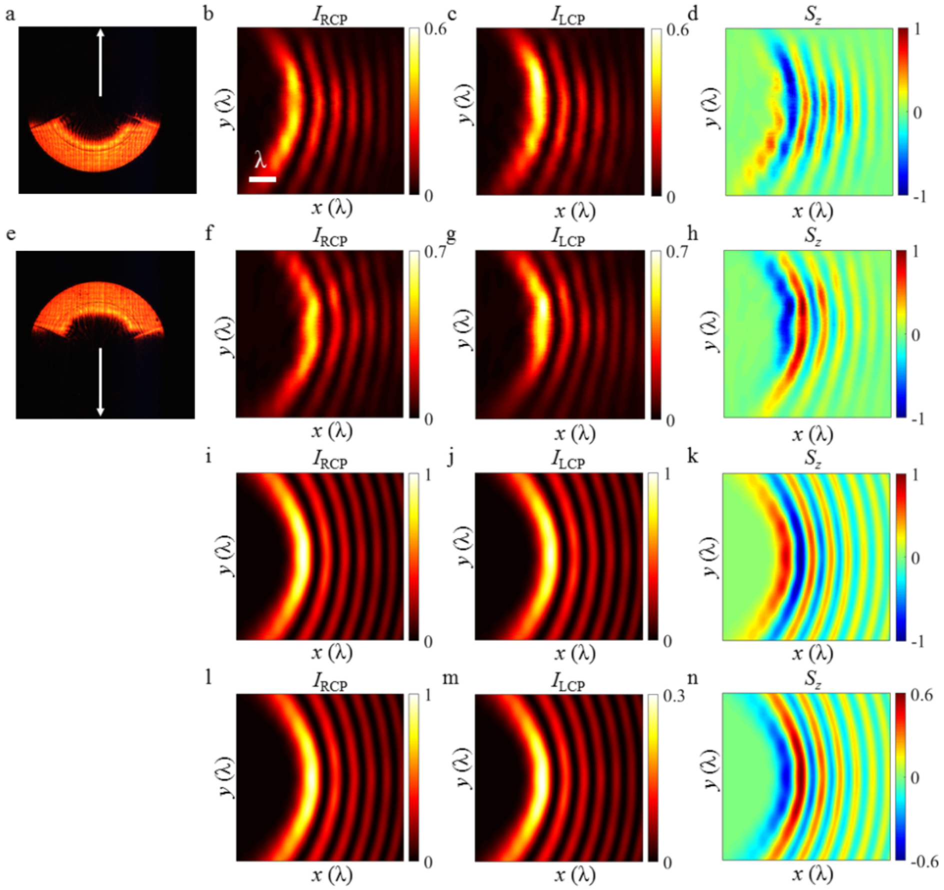


**Fig. S14. Experimental results for the SPP Weber beams. a**, The back focal plane image of the reflected beam generated with the mask in **Fig. S10**(d) and the phase diagram in **Fig. S13**(a). SPP Weber beam propagates in +y direction. **b-d**, The measured $I_{RCP}$ and $I_{LCP}$ distributions and the retrieved distribution of the $S_z$ component of the SPP Weber beam. **e-h**, The same as **b-d** for the SPP Weber beam propagating along the –y direction (generated with the mask shown in **Fig. S10**(e) the phase diagram shown in **Fig. S13**(b)). The arrows in **a** and **e** indicate the propagating directions of the generated Weber beams. The scanning step size in the experiment was 25 nm. **i-k** and **l-n**, The corresponding theoretical calculation results obtained with the vectorial diffraction theory for **b-d** and **e-h**, respectively. The distance units is the wavelength of light in vacuum.

The method for generating the SPP Airy beams can be found in [s15]. The cubic phase

$$\psi_{Airy} = -i\left\{\left(a_1 \arcsin\left(k_y/\beta\right)\right)^3 /3 + b_1 \arcsin\left(k_y/\beta\right)\right\} \tag{S97}$$

was encoded into the SLM for generation of the surface Airy beams with parameters $a_1$=0.1 and $b_1$= 15. The phase diagram shown in **Fig. S13**(c), together with the opaque mask shown in **Fig. S10**(d) were employed to generate the SPP Airy beam propagating in +*y* direction. Similarly, the phase diagram shown in **Fig.**

**S13**(d) and the opaque mask shown in **Fig. S10**(e) was employed to generate the Airy beam propagating in –*y* direction. The experimental results along with the theoretical simulations are shown in **Fig. S15**.

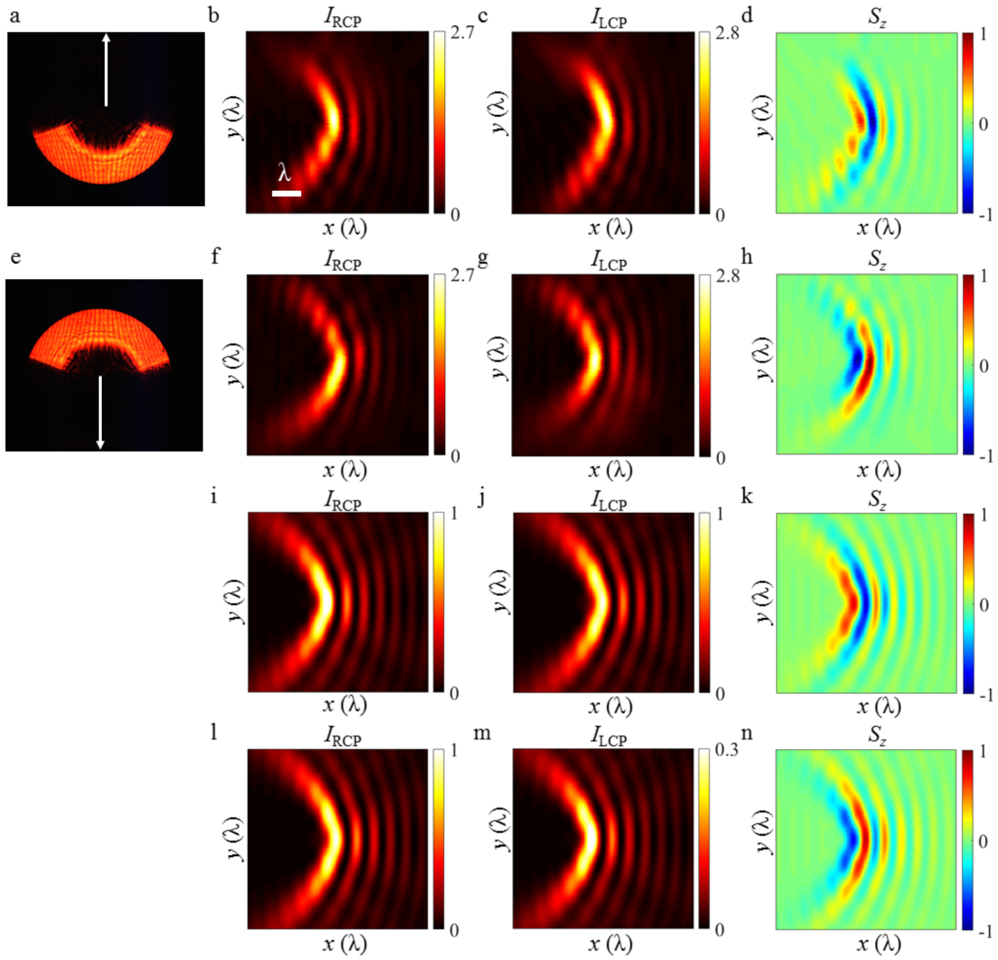


**Fig. S15. Experimental results for the SPP Airy beams. a**, The back focal plane image of the reflected beam generated with the mask in **Fig. S10**(d) and the phase diagram in **Fig. S13**(c). SPP Airy beam propagates in the +y direction. **b-d**, The measured $I_{RCP}$ and $I_{LCP}$ distributions and the retrieved distribution of the $S_z$ component of the generated SPP Airy beam. **e-h**, The same as **b-d** for an SPP Airy beam propagating in the –y direction (generated with the in **Fig. S10**(e) and the phase diagram in **Fig. S13**(d)). The scanning step size in the experiment was 25 nm. The arrows in **a** and **e** indicate the propagating directions of the generated Airy beams. **i-k** and **l-n**, The corresponding theoretical calculation results obtained with the vectorial diffraction theory for **b-d** and **e-h**, respectively. The distance units is the wavelength of light in vacuum.

## VIII. Reconstruction of the in-plane spin angular momentum components

For transverse magnetic (TM) evanescent modes, using the Maxwell's equation, the relation between the transverse electric, magnetic field components and longitudinal electric field component ($E_z$) can be expressed by Eq. (S13). Using the above relationships, the spin angular momentum components can be calculated to be:

$$\begin{cases} S_x = 2\mathrm{K}k_z \,\mathrm{Im}\left( E_z^* \dfrac{\partial E_z}{\partial y} \right) \\ S_y = 2\mathrm{K}k_z \,\mathrm{Im}\left( E_z \dfrac{\partial E_z^*}{\partial x} \right) \\ S_z = 2\mathrm{K}\,\mathrm{Im}\left( \dfrac{\partial E_z^*}{\partial x} \dfrac{\partial E_z}{\partial y} \right) \end{cases}, \tag{S.98}$$

where $\mathrm{K}=\varepsilon/4\omega\beta^2$ is a physical constant. By carefully examining the expression of spin vectors, we find that

$$\frac{\partial S_x}{\partial y} = 2\mathrm{K}k_z \,\mathrm{Im}\left( \frac{\partial E_z^*}{\partial y}\frac{\partial E_z}{\partial y} + E_z^* \frac{\partial^2 E_z}{\partial^2 y} \right) = 2\mathrm{K}k_z \,\mathrm{Im}\left( E_z^* \frac{\partial^2 E_z}{\partial^2 y} \right), \tag{S.99a}$$

and

$$\frac{\partial S_y}{\partial x} = 2\mathrm{K}k_z \,\mathrm{Im}\left( \frac{\partial E_z}{\partial x}\frac{\partial E_z^*}{\partial x} + E_z \frac{\partial^2 E_z^*}{\partial x^2} \right) = 2\mathrm{K}k_z \,\mathrm{Im}\left( E_z \frac{\partial^2 E_z^*}{\partial x^2} \right). \tag{S.99b}$$

On the other hand, the *z*-component electric field $E_z$ satisfies Helmholtz equation:

$$\nabla^2 E_z + k^2 E_z = \frac{\partial^2 E_z}{\partial x^2} + \frac{\partial^2 E_z}{\partial y^2} + \beta^2 E_z = 0. \tag{S.100}$$

Therefore, by employing the Eq. (S100), the Eq. (S99a) can be converted into

$$\frac{\partial S_x}{\partial y} = 2\mathrm{K}k_z \,\mathrm{Im}\left( -E_z^* \frac{\partial^2 E_z}{\partial x^2} - \beta^2 E_z^* E_z \right) = 2\mathrm{K}k_z \,\mathrm{Im}\left( E_z \frac{\partial^2 E_z^*}{\partial x^2} \right) = \frac{\partial S_y}{\partial x}. \tag{S.101}$$

By employing the conservation law of the spin vectors ( $\nabla \cdot \mathbf{S} = 0$ which can be deduced from Eq. (S7), one can obtain a pair of linearly partial differential equations for transverse spin vectors:

$$\frac{\partial^2 S_x}{\partial x^2} + \frac{\partial^2 S_x}{\partial y^2} = 2k_z \frac{\partial S_z}{\partial x}, \tag{S.102a}$$

$$\frac{\partial^2 S_y}{\partial x^2} + \frac{\partial^2 S_y}{\partial y^2} = 2k_z \frac{\partial S_z}{\partial y}. \tag{S.102b}$$

The transverse SAM component $S_x$ can be expressed by the Fourier expansion

$$S_x = \sum_{n=-\infty}^{\infty} \sum_{m=-\infty}^{\infty} \left( A_m \sin\frac{m\pi x}{L_x} + B_m \cos\frac{m\pi x}{L_x} \right)\left( C_n \sin\frac{n\pi y}{L_y} + D_n \cos\frac{n\pi y}{L_y} \right), \tag{S.103}$$

where $L_x$ and $L_y$ are boundary condition parameters. $A_m$, $B_m$, $C_m$ and $D_m$ are constants to be determined. From Eq. (S.102a) and Eq. (S.103), we can get that:

$$-\sum_{n=-\infty}^{\infty} \sum_{m=-\infty}^{\infty} \lambda_{mn} \left( A_m \sin\frac{m\pi x}{L_x} + B_m \cos\frac{m\pi x}{L_x} \right)\left( C_n \sin\frac{n\pi y}{L_y} + D_n \cos\frac{n\pi y}{L_y} \right) = 2k_z \frac{\partial S_z}{\partial x}, \tag{S.104}$$

where $\lambda_{mn}=\left(\frac{m\pi}{L_x}\right)^2+\left(\frac{n\pi}{L_y}\right)^2$ is the eigenvalue. The expansion coefficients can be determined through the Fourier integral of equation (S.104). Omitting the complex mathematical process, we obtain that $S_x$ can be solved to be：

$$\begin{cases} S_x=\sum_{n=-\infty}^{\infty}\sum_{m=-\infty}^{\infty}\begin{pmatrix} A'_m\sin\frac{m\pi x}{L_x}\sin\frac{n\pi y}{L_y}+B'_m\sin\frac{m\pi x}{L_x}\cos\frac{n\pi y}{L_y} \\ +C'_m\cos\frac{m\pi x}{L_x}\sin\frac{n\pi y}{L_y}+D'_m\cos\frac{m\pi x}{L_x}\cos\frac{n\pi y}{L_y}\end{pmatrix} \\ A'_m=-\frac{1}{4L_xL_y\lambda_{mn}}\int_0^{L_x}\int_0^{L_y}2k_z\frac{\partial S_z}{\partial x}\sin\frac{m\pi x}{L_x}\sin\frac{n\pi y}{L_y}dxdy \\ B'_m=-\frac{1}{4L_xL_y\lambda_{mn}}\int_0^{L_x}\int_0^{L_y}2k_z\frac{\partial S_z}{\partial x}\sin\frac{m\pi x}{L_x}\cos\frac{n\pi y}{L_y}dxdy \\ C'_m=-\frac{1}{4L_xL_y\lambda_{mn}}\int_0^{L_x}\int_0^{L_y}2k_z\frac{\partial S_z}{\partial x}\cos\frac{m\pi x}{L_x}\sin\frac{n\pi y}{L_y}dxdy \\ D'_m=-\frac{1}{4L_xL_y\lambda_{mn}}\int_0^{L_x}\int_0^{L_y}2k_z\frac{\partial S_z}{\partial x}\cos\frac{m\pi x}{L_x}\cos\frac{n\pi y}{L_y}dxdy \end{cases}. \tag{S.105}$$

In a similar manner, from Eq. (S.102b), $S_y$ can be expressed as:

$$\begin{cases} S_y=\sum_{n=-\infty}^{\infty}\sum_{m=-\infty}^{\infty}\begin{pmatrix} A''_m\sin\frac{m\pi x}{L_x}\sin\frac{n\pi y}{L_y}+B''_m\sin\frac{m\pi x}{L_x}\cos\frac{n\pi y}{L_y} \\ +C''_m\cos\frac{m\pi x}{L_x}\sin\frac{n\pi y}{L_y}+D''_m\cos\frac{m\pi x}{L_x}\cos\frac{n\pi y}{L_y}\end{pmatrix} \\ A''_m=-\frac{1}{4L_xL_y\lambda_{mn}}\int_0^{L_x}\int_0^{L_y}2k_z\frac{\partial S_z}{\partial y}\sin\frac{m\pi x}{L_x}\sin\frac{n\pi y}{L_y}dxdy \\ B''_m=-\frac{1}{4L_xL_y\lambda_{mn}}\int_0^{L_x}\int_0^{L_y}2k_z\frac{\partial S_z}{\partial y}\sin\frac{m\pi x}{L_x}\cos\frac{n\pi y}{L_y}dxdy \\ C''_m=-\frac{1}{4L_xL_y\lambda_{mn}}\int_0^{L_x}\int_0^{L_y}2k_z\frac{\partial S_z}{\partial y}\cos\frac{m\pi x}{L_x}\sin\frac{n\pi y}{L_y}dxdy \\ D''_m=-\frac{1}{4L_xL_y\lambda_{mn}}\int_0^{L_x}\int_0^{L_y}2k_z\frac{\partial S_z}{\partial y}\cos\frac{m\pi x}{L_x}\cos\frac{n\pi y}{L_y}dxdy \end{cases}. \tag{S.106}$$

Through the measured longitudinal SAM component $S_z$, the transverse SAM components can be obtained from Eq. (S.105) and Eq. (S.106), and a complete photonic spin vector can be constructed.

In addition, we can employ the symmetry of surface electromagnetic modes to simplify the calculation further. Taking the surface Bessel mode for example, $S_z$ is mirror symmetric with respect to *x*, *y*-axes, which can be expressed as $\hat{m}_xS_z=S_z$ and $\hat{m}_yS_z=S_z$, where $\hat{m}_i$ is the mirror operator with respect to *i*-axis in Cartesian coordinates. Noted that the *i*-coordinate is mirror antisymmetric with respect to *i*-axis is mirror symmetric with respect to another axis, so that we have $\hat{m}_xx=-x$ and $\hat{m}_yx=x$. Thus, we can get the following relationships:

$$\hat{m}_x\frac{\partial S_z}{\partial x}=-\frac{\partial S_z}{\partial x}\quad \hat{m}_y\frac{\partial S_z}{\partial x}=\frac{\partial S_z}{\partial x}, \tag{S.107}$$

which can be utilized to simplify the Eqs. (S105) and (S106):

$$\begin{cases} S_x = \sum_{n=-\infty}^{\infty} \sum_{m=-\infty}^{\infty} \left( A_m \sin \frac{m\pi x}{L_x} \cos \frac{n\pi y}{L_y} \right) \\ A_m = -\frac{1}{4L_x L_y \lambda_{mn}} \int_0^{L_x} \int_0^{L_y} 2k_z \frac{\partial S_z}{\partial x} \sin \frac{m\pi x}{L_x} \cos \frac{n\pi y}{L_y} dxdy \end{cases}, \tag{S.108a}$$

$$\begin{cases} S_y = \sum_{n=-\infty}^{\infty} \sum_{m=-\infty}^{\infty} \left( B_m \cos \frac{m\pi x}{L_x} \sin \frac{n\pi y}{L_y} \right) \\ B_m = -\frac{1}{4L_x L_y \lambda_{mn}} \int_0^{L_x} \int_0^{L_y} 2k_z \frac{\partial S_z}{\partial y} \cos \frac{m\pi x}{L_x} \sin \frac{n\pi y}{L_y} dxdy \end{cases}. \tag{S.108b}$$

Through this method, we reconstructed from the experimental measurements the in-plane SAM components for the four structured waves as shown in **Figs. S16-19**, where the results obtained from the experiment match well with the theoretical simulations.

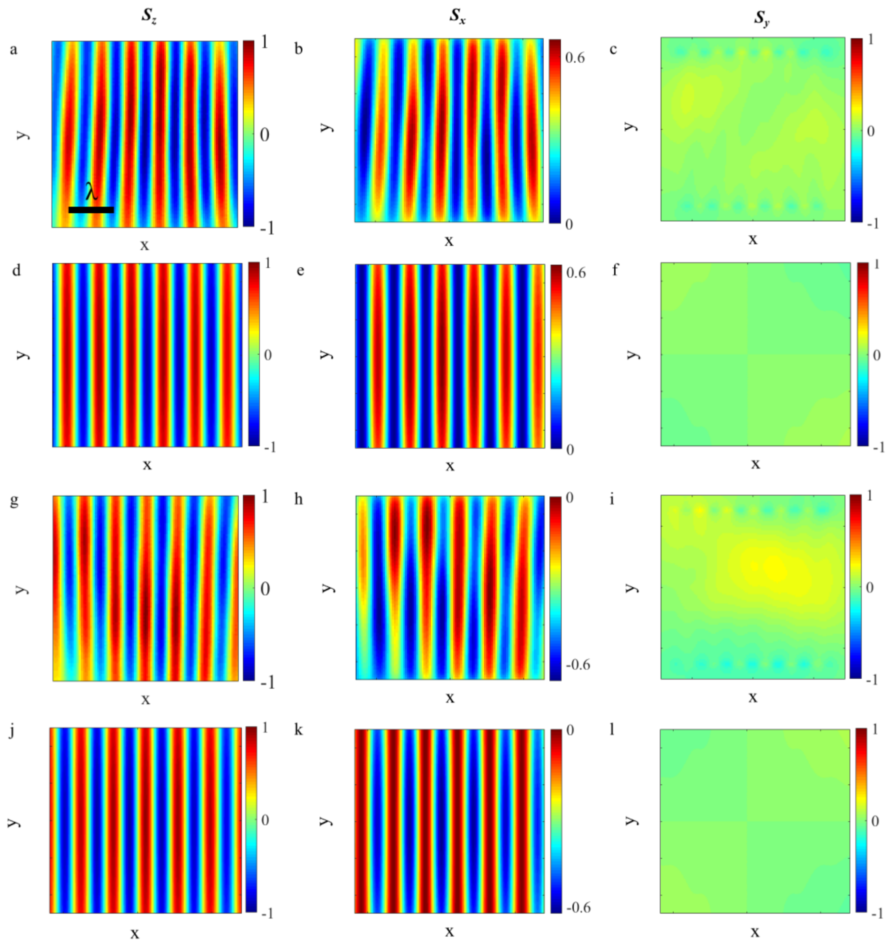


**Fig. S16. Measured and simulated SAM components for the SPP Cosine beams. a-c** and **g-i,** experimentally obtained and **d-f** and **j-l** theoretical SAM components **a**,**d**,**g**,**j** $S_z$, **b**,**e**,**h**,**k** $S_x$ and **c**,**f**,**i**,**l** $S_y$ for the SPP Cosine beams propagating in **a-f** +y and **g-l** −y direction. The scale bar is the wavelength of light in vacuum.

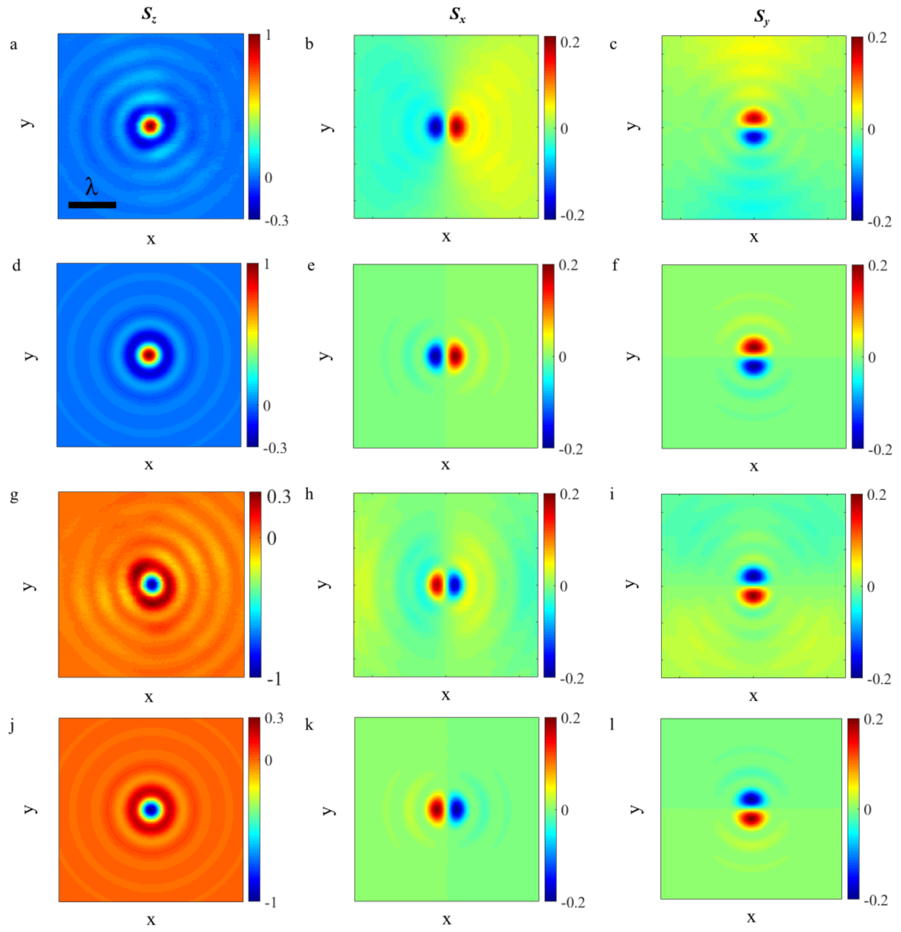


**Fig. S17. Measured and simulated SAM components for the SPP Bessel beams. a-c** and **g-i,** experimentally obtained and **d-f** and **j-l** theoretical SAM components **a**,**d**,**g**,**j** $S_z$, **b**,**e**,**h**,**k** $S_x$ and **c**,**f**,**i**,**l** $S_y$ for the SPP Bessel beams with vortex topological charge **a-f** +1 and **g-l** −1. The scale bar is the wavelength of light in vacuum.

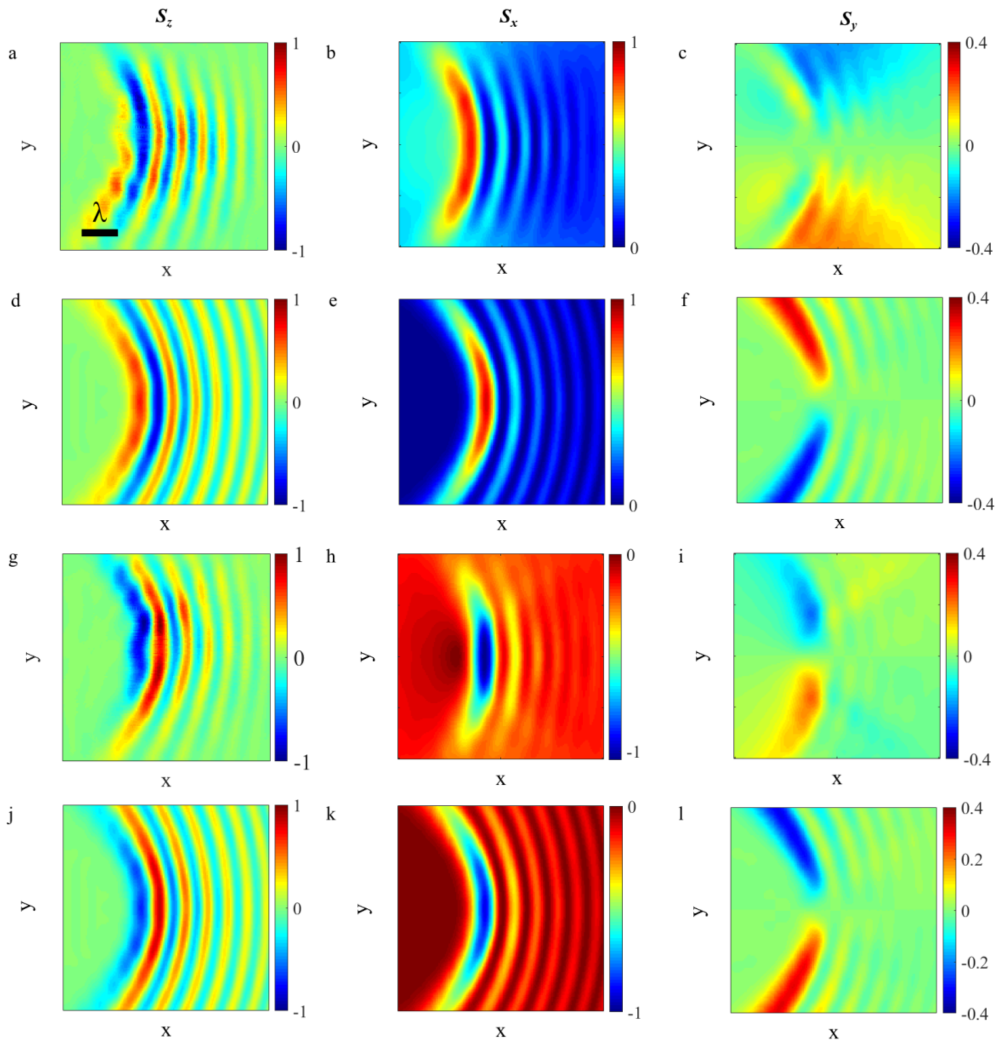


**Fig. S18. Measured and simulated SAM components for the SPP Weber beams. a-c** and **g-i,** experimentally obtained and **d-f** and **j-l** theoretical SAM components **a**,**d**,**g**,**j** $S_z$, **b**,**e**,**h**,**k** $S_x$ and **c**,**f**,**i**,**l** $S_y$ for the SPP Weber beams propagating in **a-f +P** and **g-l −P** direction. The scale bar is the wavelength of light in vacuum.

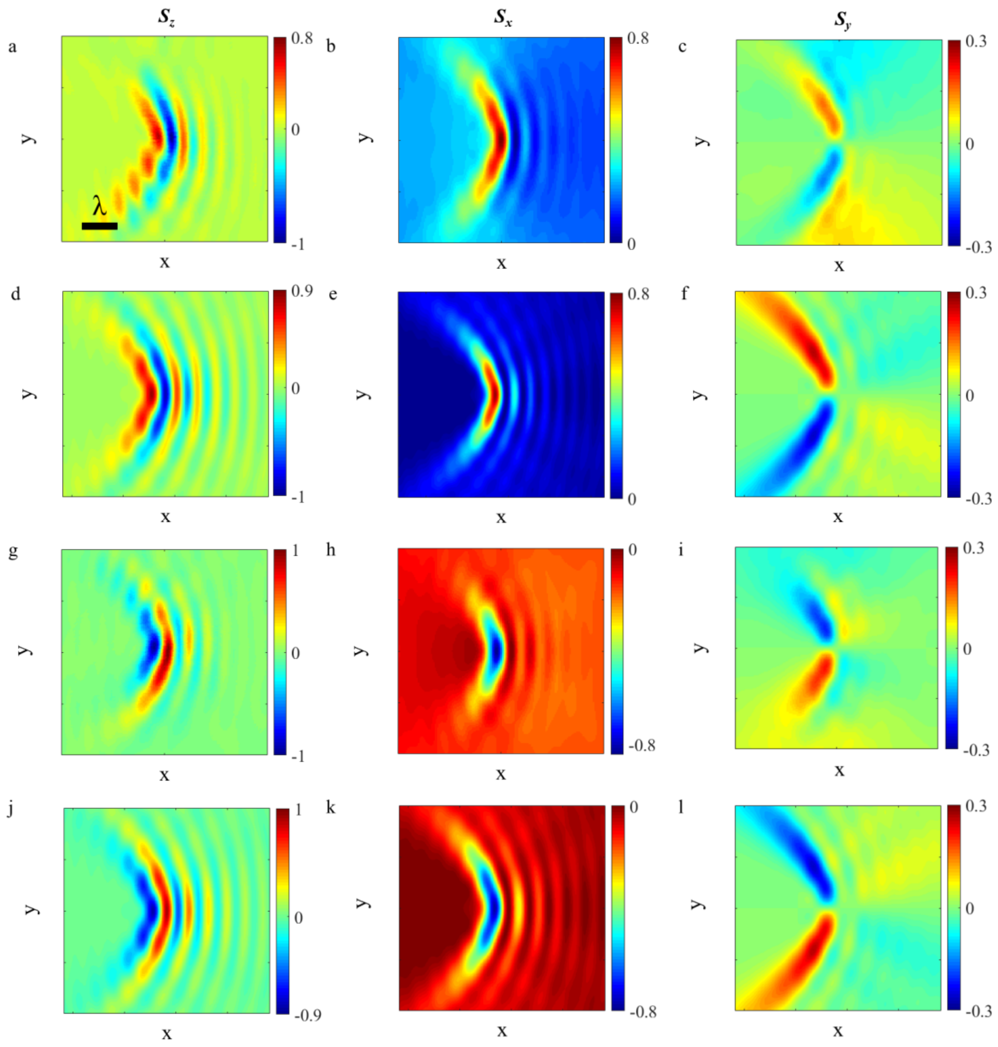


**Fig. S19. Measured and simulated SAM components for the SPP Airy beams. a-c** and **g-i**, experimentally obtained and **d-f** and **j-l** theoretical SAM components **a,d,g,j** $S_z$, **b,e,h,k** $S_x$ and **c,f,i,l** $S_y$ for the SPP Airy beams propagating in **a-f** +**P** and **g-l** −**P** direction. The scale bar is the wavelength of light in vacuum.

## SI References


s1. Jackson J. D., (1999) Classical Electrodynamics, *New York: Wiley* p19.

s2. Barnett Stephen M., (2014 ) Optical Dirac equation. *New Journal of Physics* 16:093008.

s3. Bialynicki-Birula I., and Bialynicka-Birula Z., (2013) The role of the Riemann–Silberstein vector in classical and quantum theories of electromagnetism. *J. Phys. A: Math. Theor.* 46:053001.

s4. Elbistan, M., Horváthy, P. A., Zhang P.-M., (2017) Duality and helicity: the photon wave function approach, *Physics Letters A* 381:2375–2379.

s5. Auld B. A., (1973) Acoustic fields and waves in solids Volume I, *New York: John Wiley & Sons INC*. p123.

s6. Mustafa E., and Cohen J. M., (1987) Hertz and Debye potentials and electromagnetic fields in general relativity. *Class. Quantum Grav.* 4:1623.

s7. Wang R., *et al.* (2017) Bloch surface waves confined in one dimension with a single polymeric nanofibre, *Nature Communications* 8:14330.

s8. Shen Shun-Qing, (2012) Topological Insulators: Dirac Equation in Condensed Matters, *Springer-Verlag Berlin Heidelberg* p181.

s10. Li Chun-Fang, (2009) Spin and orbital angular momentum of a class of nonparaxial light beams having a globally defined polarization. *Phys. Rev. A* 80:063814.

s11. Bliokh K. Y., Alonso Miguel A., Ostrovskaya Elena A., and Aiello A., (2010) Angular momenta and spin-orbit interaction of nonparaxial light in free space. *Phys. Rev. A* 82:063825.

s12. Bliokh K. Y., Gorodetski Y., Kleiner V., and Hasman E., (2008) Coriolis Effect in Optics: Unified Geometric Phase and Spin-Hall Effect. *Phys. Rev. Lett.* 101:030404.

s13. Bliokh K. Y., Niv A., Kleiner V., and Hasman E., (2008) Geometrodynamics of spinning light, *Nature Photonics* 2:748-753.

s14. Duval C., Horváth Z., Horváthy P.A., (2007) Geometrical spinoptics and the optical Hall effect. *Journal of Geometry and Physics* 57:925–941.

s15. Kou Shan Shan, *et al.* (2016) On-chip photonic Fourier transform with surface plasmon polaritons, *Light: Science & Applications* 5:e16034.

s16. Yang, A.-P., Du, L.-P., Meng, F.-F., and Yuan, X.-C., Optical transverse spin coupling through a plasmonic nanoparticle for particle-identifcation and feld-mapping. *Nanoscale* **10**, 9286–9291 (2018).

s17. Novotny L., Hecht B., and Keller O., (2012) Principles of nano-optics, *Cambridge University Press* p81.
